# Supplementary material for: Neither “lumpers” nor “splitters”: A global revision of Flabellinidae s.l. nudibranchs (Gastropoda: Heterobranchia: Nudibranchia)
Source: PLoS One. 2026 May 20;21(5):e0347759. doi: 10.1371/journal.pone.0347759 (PMC13189321; doi:10.1371/journal.pone.0347759)
Supplement: S2 Table — (DOCX) [file pone.0347759.s003.docx]

**Table S2. Amplification and sequencing primers and PCR conditions.**

| Marker | Primers | PCR conditions | References |
| --- | --- | --- | --- |
| Cytochrome *c* oxidase  subunit I | **LCO1490** GGTCAACAAATCATAAAGATATTGG **HCO2198** TAAACTTCAGGGTGACCAAAAAATCA | 5 min – 95 °C, 35x [15 s – 95 °C, 30 s – 45 °C, 1 min – 72 °C], 7 min – 72 °C | Folmer et al., 1994 |
| 16S rRNA | **16Sar-L**  CGCCTGTTTATCAAAAACAT  **16S BRh**  CCGGTCTGAACTCAGATCACGT | 5 min – 95 °C, 35x [15 s – 95 °C, 30 s – 52 °C, 1 min – 72 °C], 7 min – 72 °C | Palumbi et al., 1991 |
| Histone H3 | **H3AF**  ATGGCTCGTACCAAGCAGACVGC **H3AR**  ATATCCTTRGGCATRATRGTGAC | 5 min – 95 °C, 35x [15 s – 95 °C, 30 s – 50 °C, 1 min – 72 °C], 7 min – 72 °C | Colgan et al., 1998 |
| 28S rRNA | **28S C1**  ACCCGCTGAATTTAAGCAT  **28S C2**  TGAACTCTCTCTTCAAAGTTCTTTTC | 5 min – 95 °C, 35x [30 s – 95 °C, 30 s – 50 °C, 1 min –  72 °C], 7 min – 72 °C | Dayrat et al. 2001; Lê et al, 1993 |

**References:**

1. Colgan, D. J., McLauchlan, A., Wilson, G. D., Livingston, S. P., Edgecombe, G. D., Macaranas, J., Cassis G., & Gray, M. R. (1998). Histone H3 and U2 snRNA DNA sequences and arthropod molecular evolution. Australian Journal of Zoology, 46(5), 419-437.
2. Dayrat, B., Tillier, A., Lecointre, G., & Tillier, S. (2001). New clades of euthyneuran gastropods (Mollusca) from 28S rRNA sequences. Molecular phylogenetics and evolution, 19(2), 225-235.
3. Folmer, O., Hoeh, W. R., Black, M. B., & Vrijenhoek, R. C. (1994). Conserved primers for PCR amplification of mitochondrial DNA from different invertebrate phyla. Molecular Marine Biology and Biotechnology, 3(5), 294-299.
4. Palumbi S.R., Kessing B., & Martin A. (1991). The Simple Fool’s Guide to PCR, Version 2 edition. Department of Zoology, University of Hawaii, Honolulu.
5. Vân Le, H. L., Lecointre, G., & Perasso, R. (1993). A 28S rRNA-based phylogeny of the gnathostomes: first steps in the analysis of conflict and congruence with morphologically based cladograms. Molecular phylogenetics and evolution, 2(1), 31-51.
