# Supplementary figures and images for: Neither “lumpers” nor “splitters”: A global revision of Flabellinidae s.l. nudibranchs (Gastropoda: Heterobranchia: Nudibranchia)
Source: PLoS One. 2026 May 20;21(5):e0347759. doi: 10.1371/journal.pone.0347759 (PMC13189321; doi:10.1371/journal.pone.0347759)

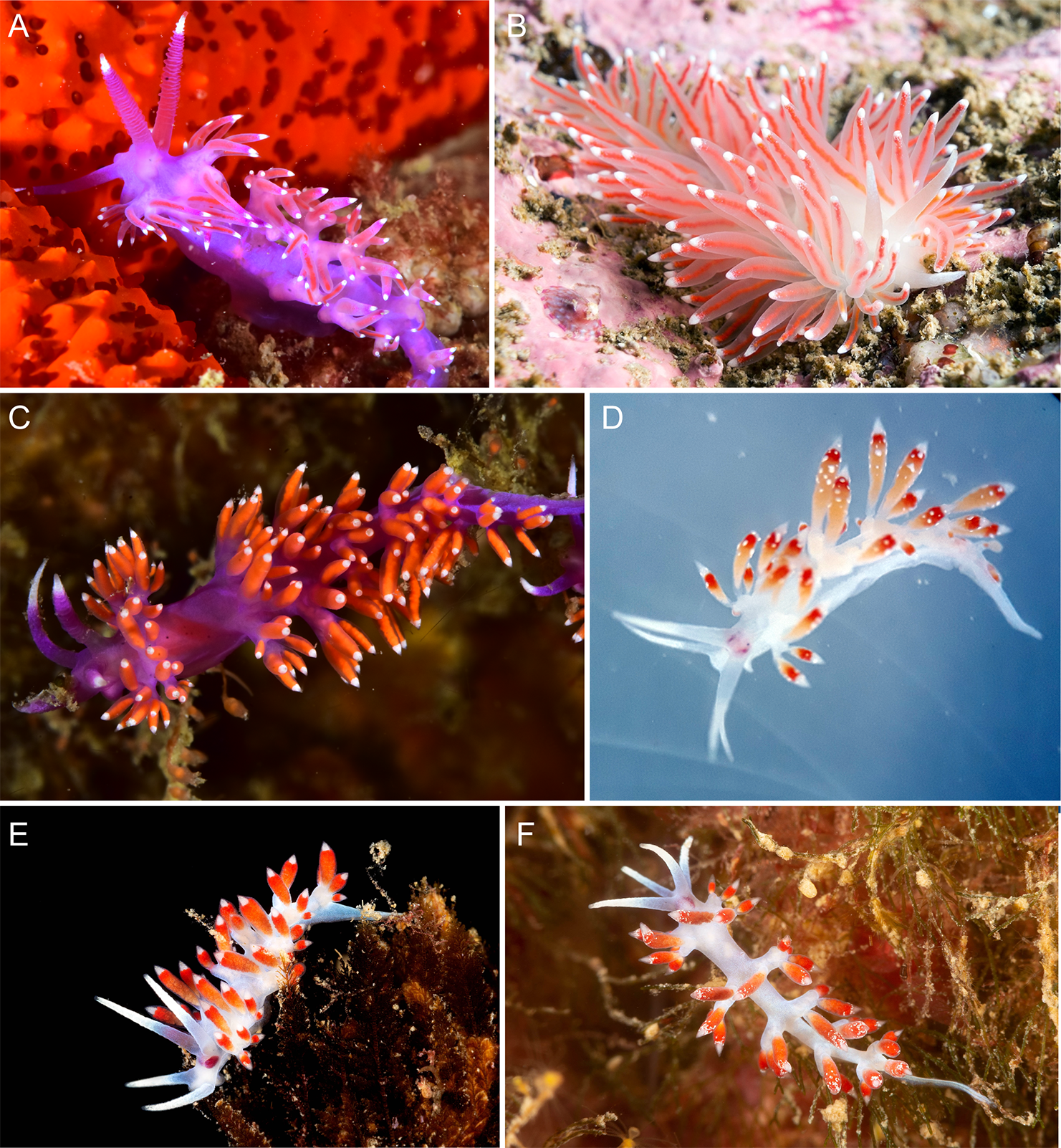

Supplement: S2 Fig — A – Flabellina affinis. B – Flabellina pellucida. C – Flabellina ischitana. D – Flabellina gaditana. E, F – Flabellina cavolini. Photo credits: A, C – Manuel Martínez Chacón. B – Tatiana Antokhina. D – Sául Patiño Gómez. E, F – Enric Madrenas. (TIF) [file pone.0347759.s008.tif]

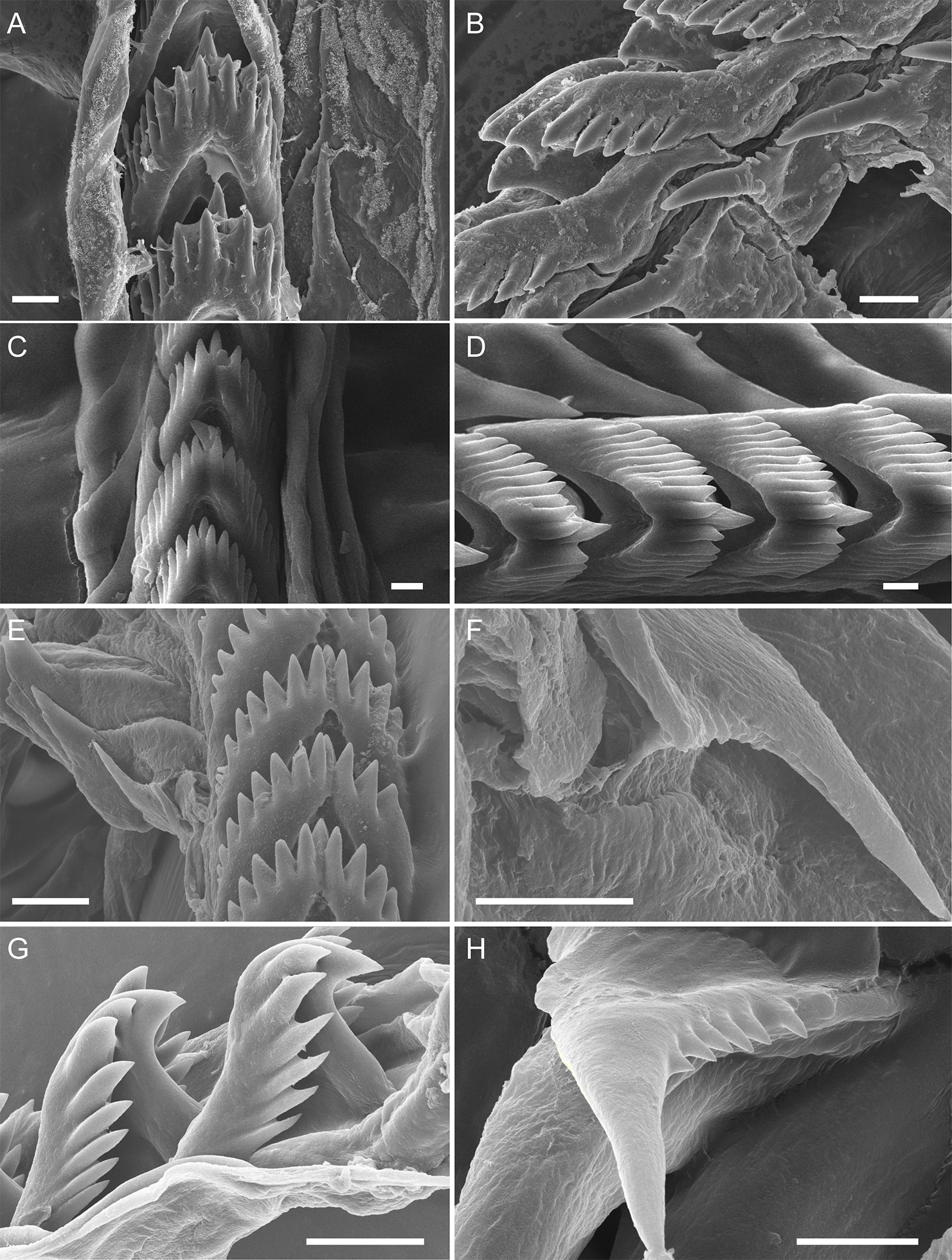

Supplement: S3 Fig — A, B – Flabellina affinis, MNCN15.05/98993. C, D – Flabellina pellucida, IEIn1 (C) and IEFp8 (D). E, F – Flabellina cavolini, MNCN15.05/98991. G, H – Flabellina ischitana, MNCN15.05/98990. Scale bars: A–E, G = 10 µm. F = 5 µm. H = 3 µm. (TIF) [file pone.0347759.s009.tif]

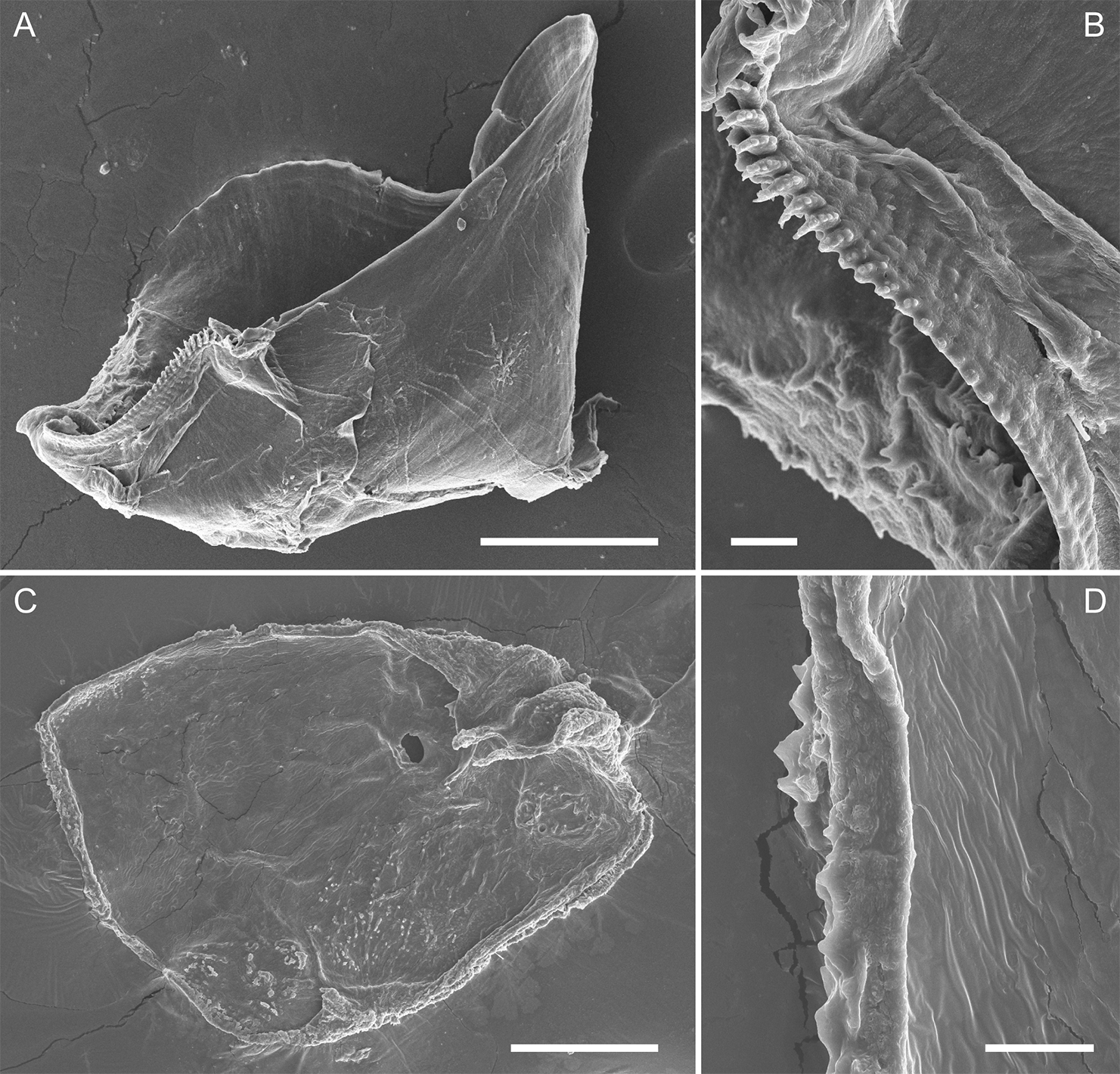

Supplement: S4 Fig — A, B – Flabellina ischitana, MNCN15.05/98990, jaw body (A) and masticatory border (B). C, D – Flabellina cavolini, MNCN15.05/98991, jaw body (C) and masticatory border (D). Scale bars: A, C = 100 µm. B, D = 10 µm. (TIF) [file pone.0347759.s010.tif]

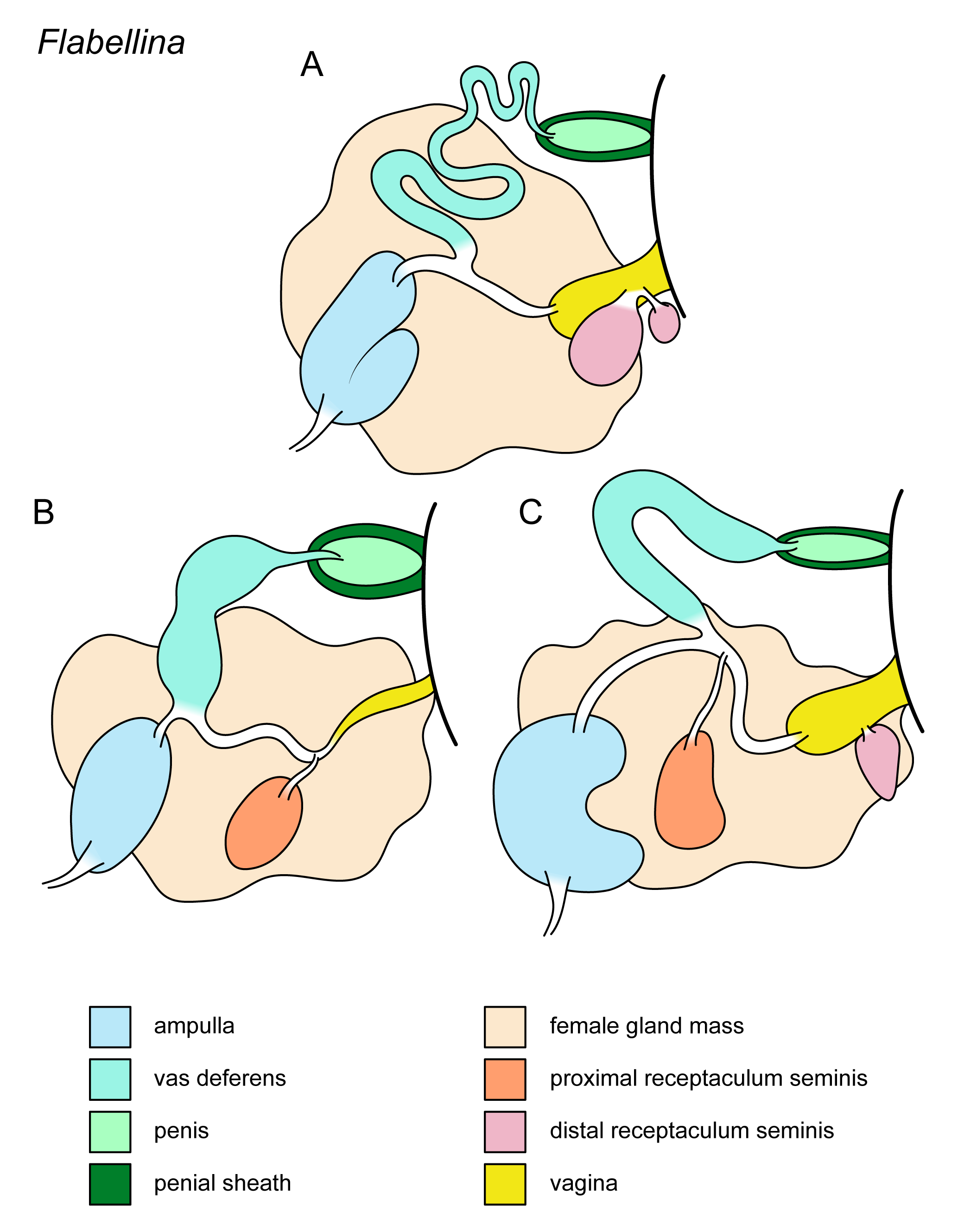

Supplement: S5 Fig — A – Flabellina affinis. B – Flabellina cavolini. C – Flabellina funeka. (TIF) [file pone.0347759.s011.tif]

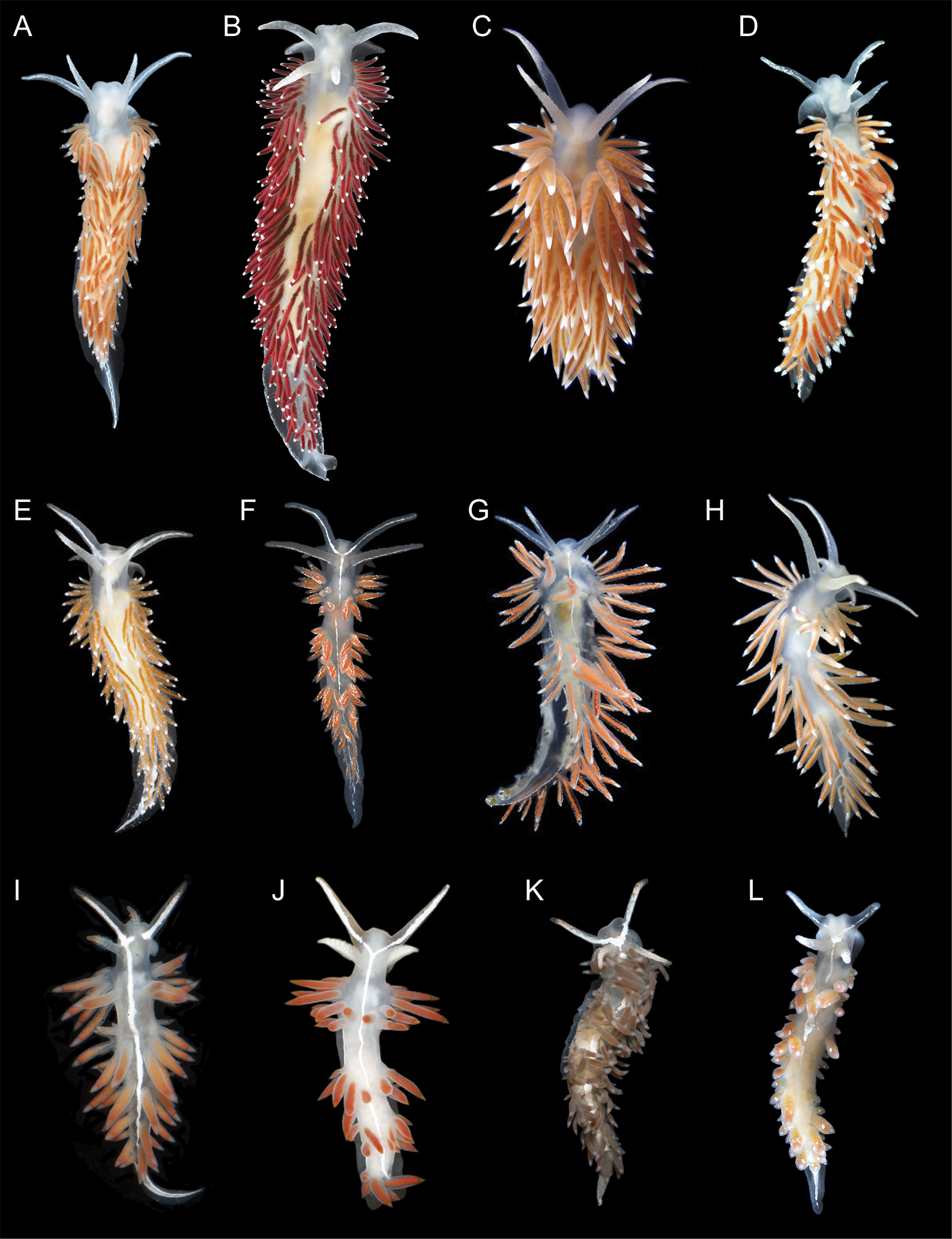

Supplement: S6 Fig — A – Coryphella verrucosa ZMMU WS14967. B – Coryphella sanamyanae ZMMU WS14987. C – C. nobilis, voucher not specified. D – C. amabilis ZMMU WS14957. E – Coryphella orjani ZMBN130734. F – C. chriskaugei ZMBN125918. G – C. lineata ZMBN127566. H – C. browni ZMBN126001. I – Coryphella sp. 2 CPIC1639. J – C. trilineata CAS218410. K – C. cooperi CPIC1646. L – C. athadona, ZMMU WS14999. Photo credits: A, B, D, L – Tatiana Antokhina, Yury Deart. C – Sergey Gorin. E–H – Manuel Malaquias. I – Ángel Valdés. J – Brenna Green. K – Craig Hoover. (TIF) [file pone.0347759.s012.tif]

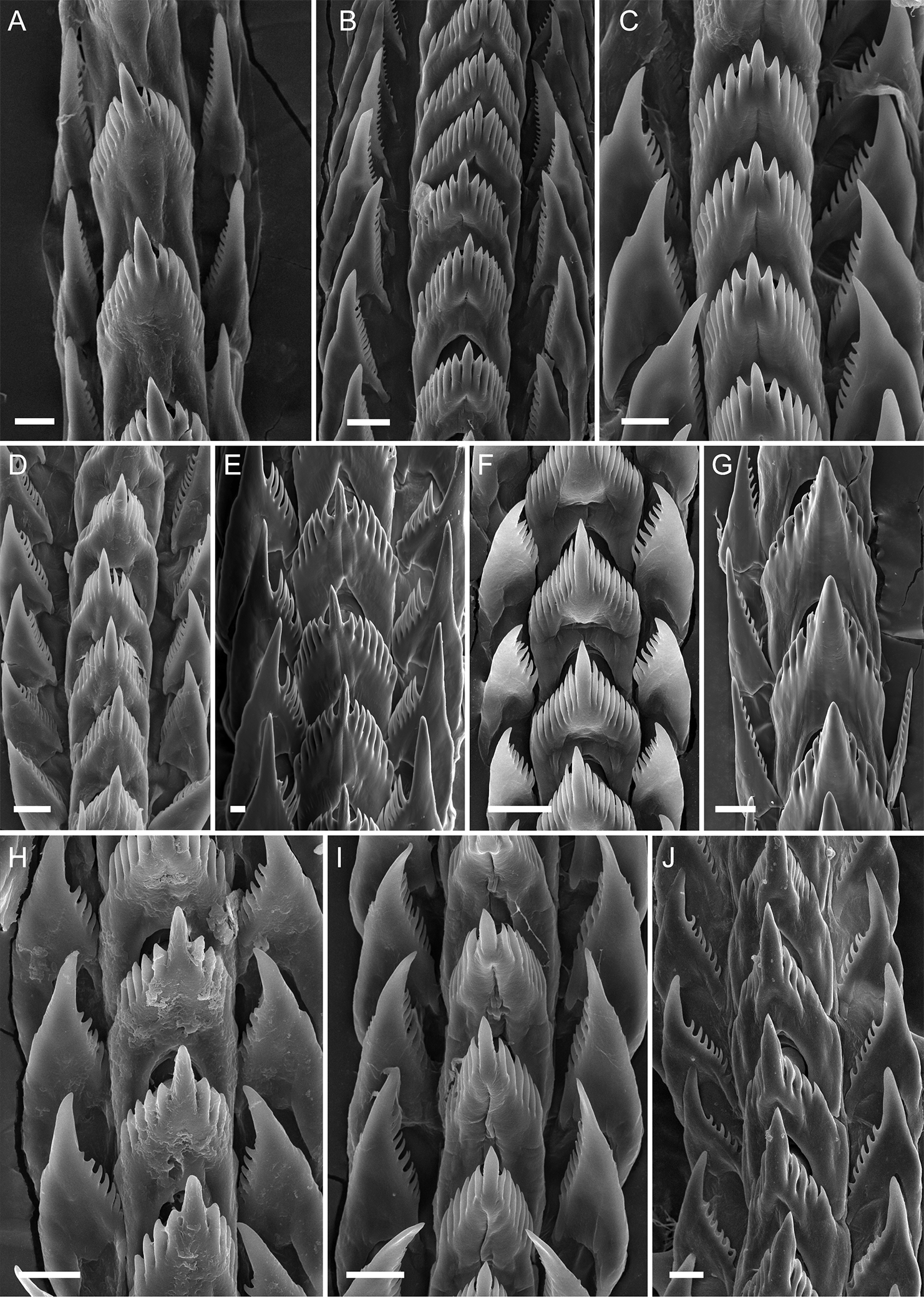

Supplement: S7 Fig — A – Coryphella verrucosa, ZMMU WS14451. B – Coryphella nobilis, ZMMU WS14379. C – Coryphella sanamyanae, ZMMU WS14416. D – Coryphella alexanderi, MIMB42469. E – Coryphella trophina, ZMMU WS14405. F – Coryphella gracilis, ZMMU WS14915. G – Coryphella falklandica, ZIN N9. H – Coryphella sp. 2, CPIC880. I – Coryphella cooperi, CPIC1646. J – Coryphella athadona, ZMMU WS14424. Scale bars: A–F, H, I = 30 µm. G = 50 µm. J = 20 µm. (TIF) [file pone.0347759.s013.tif]

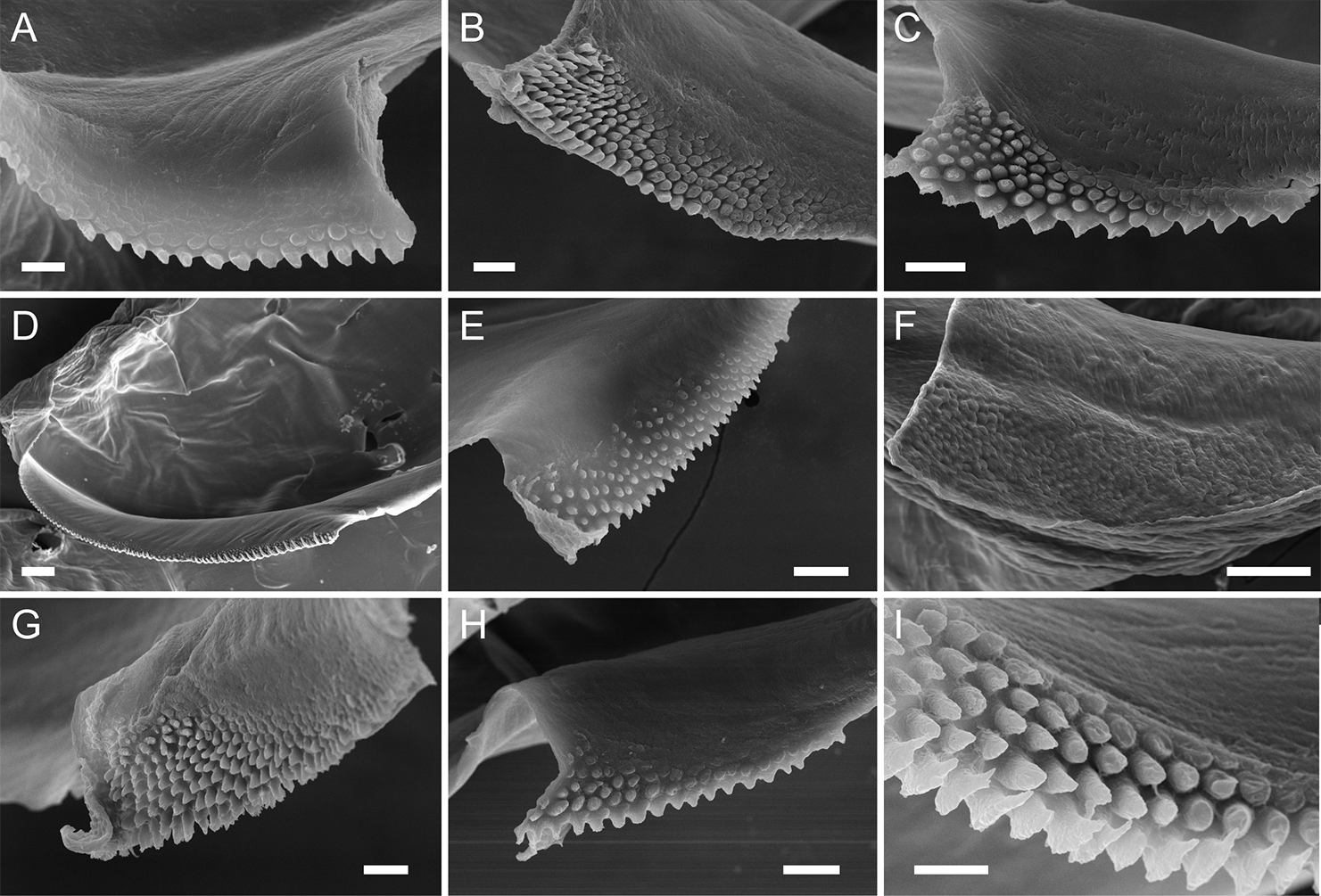

Supplement: S8 Fig — A – Coryphella verrucosa, ZMMU WS14440. B – Coryphella nobilis, MIMB40023. C – Coryphella sanamyanae, ZMMU WS14417. D – Coryphella trophina, ZMMU WS14435. E – Coryphella gracilis, ZMMU WS14914. F – Coryphella falklandica, ZIN N9. G – Coryphella alexanderi, MIMB42468. H – Coryphella sp. 2, CPIC880. I – Coryphella cooperi, CPIC1646. Scale bars: A, F, I = 50 µm. B, C, E, G, H = 30 µm. D = 100 µm. (TIF) [file pone.0347759.s014.tif]

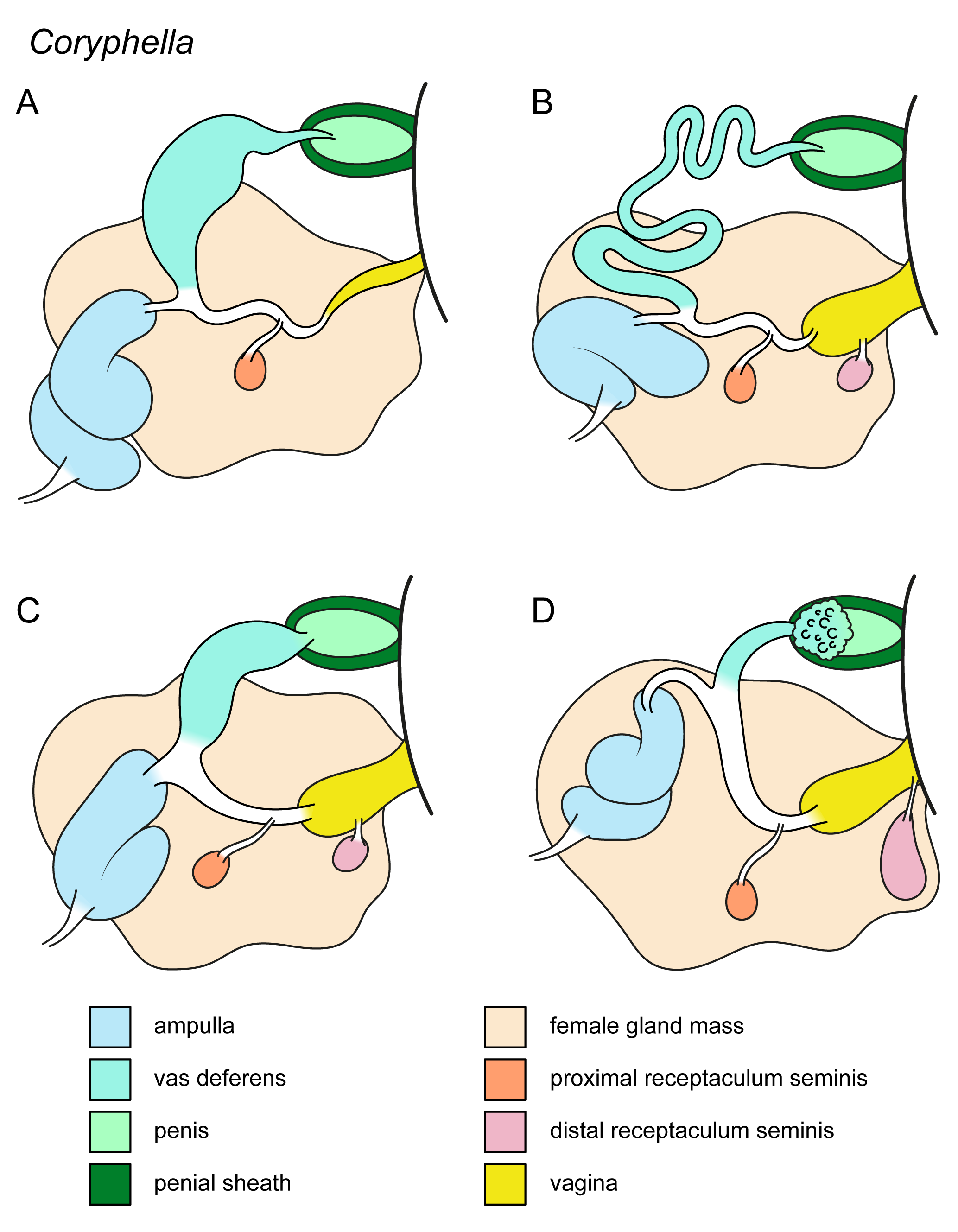

Supplement: S9 Fig — A – Coryphella falklandica. B – Coryphella gracilis. C – Coryphella verrucosa. D – Coryphella verta. (TIF) [file pone.0347759.s015.tif]

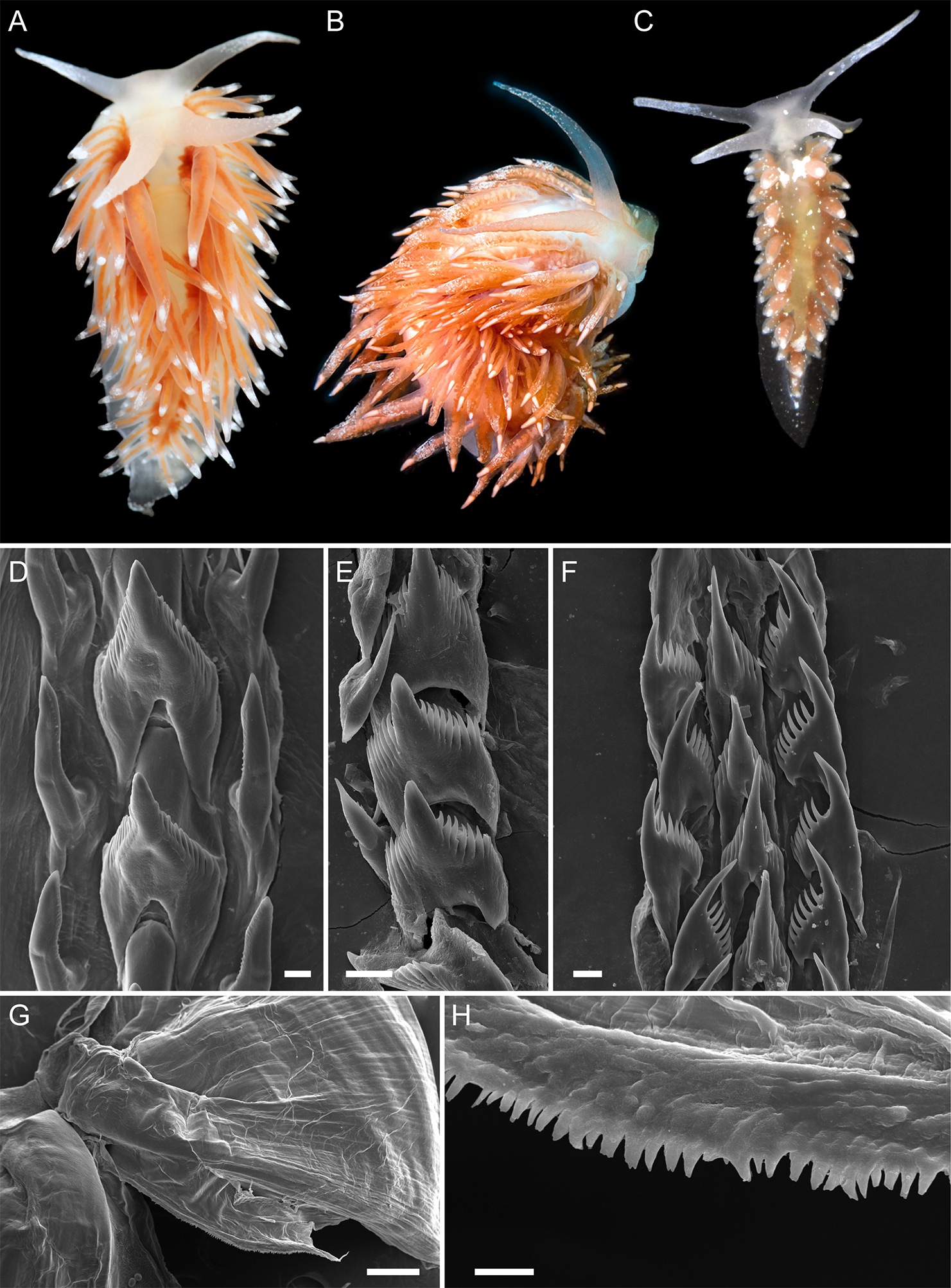

Supplement: S10 Fig — A – Chlamylla intermedia, BBS23017. B – Chlamylla borealis orientalis, Sh-1. C – Chlamylla islandica, BBS24116. D – Chlamylla intermedia, voucher not specified, radula. E – Chlamylla islandica, BBS24116, radula. F – Chlamylla cf. polaris, abyssal depth of Sea of Japan, MIMB49484, radula. G, H – Chlamylla intermedia, voucher not specified, radula. Photo credits: A, C – Irina Ekimova; B – Andrey Shpatak. Scale bars: D–F, H = 20 µm. G = 200 µm. (TIF) [file pone.0347759.s016.tif]

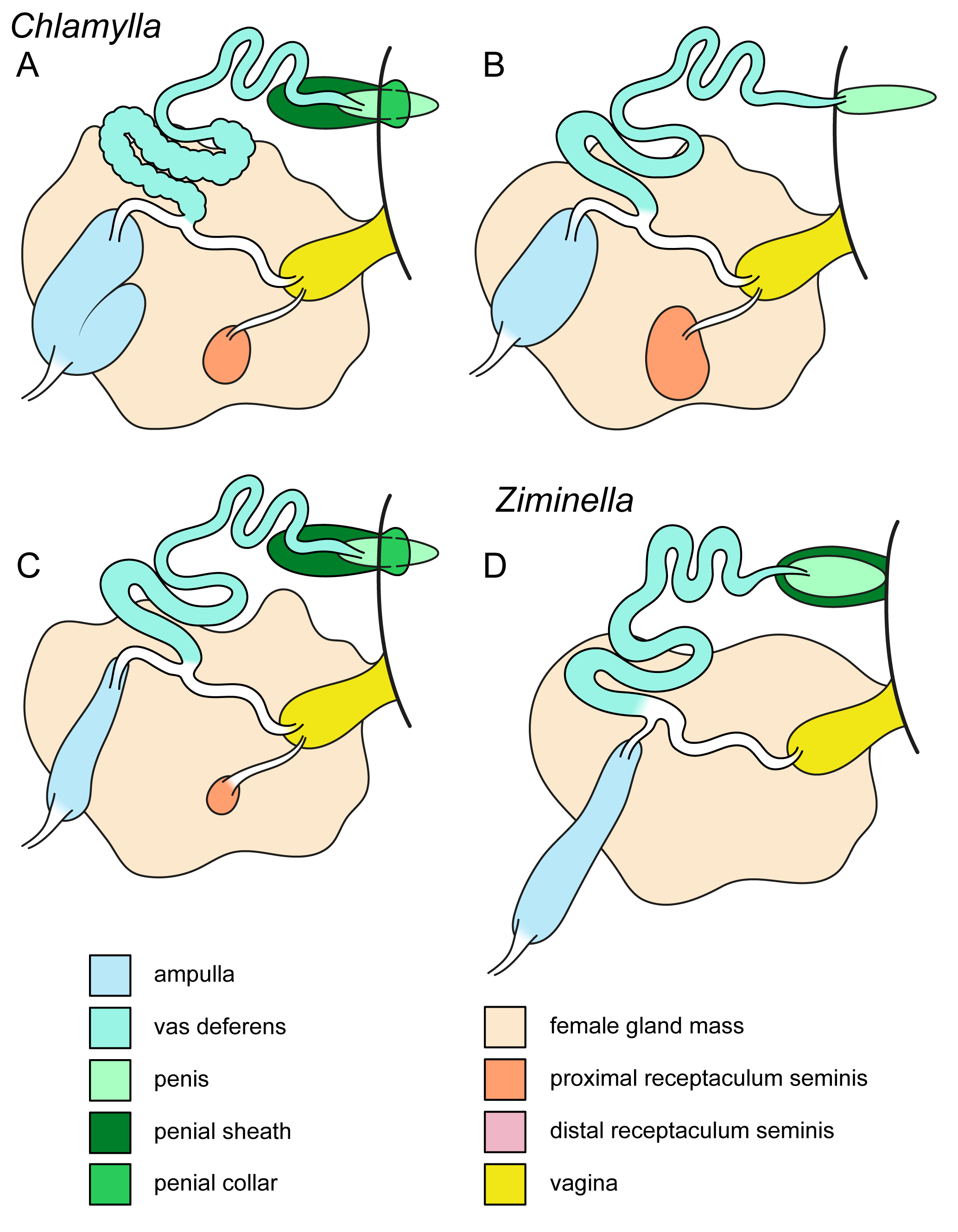

Supplement: S11 Fig — A – Chlamylla intermedia. B – Chlamylla islandica. C – Chlamylla polaris. D – Ziminella vrijenhoeki. (TIF) [file pone.0347759.s017.tif]

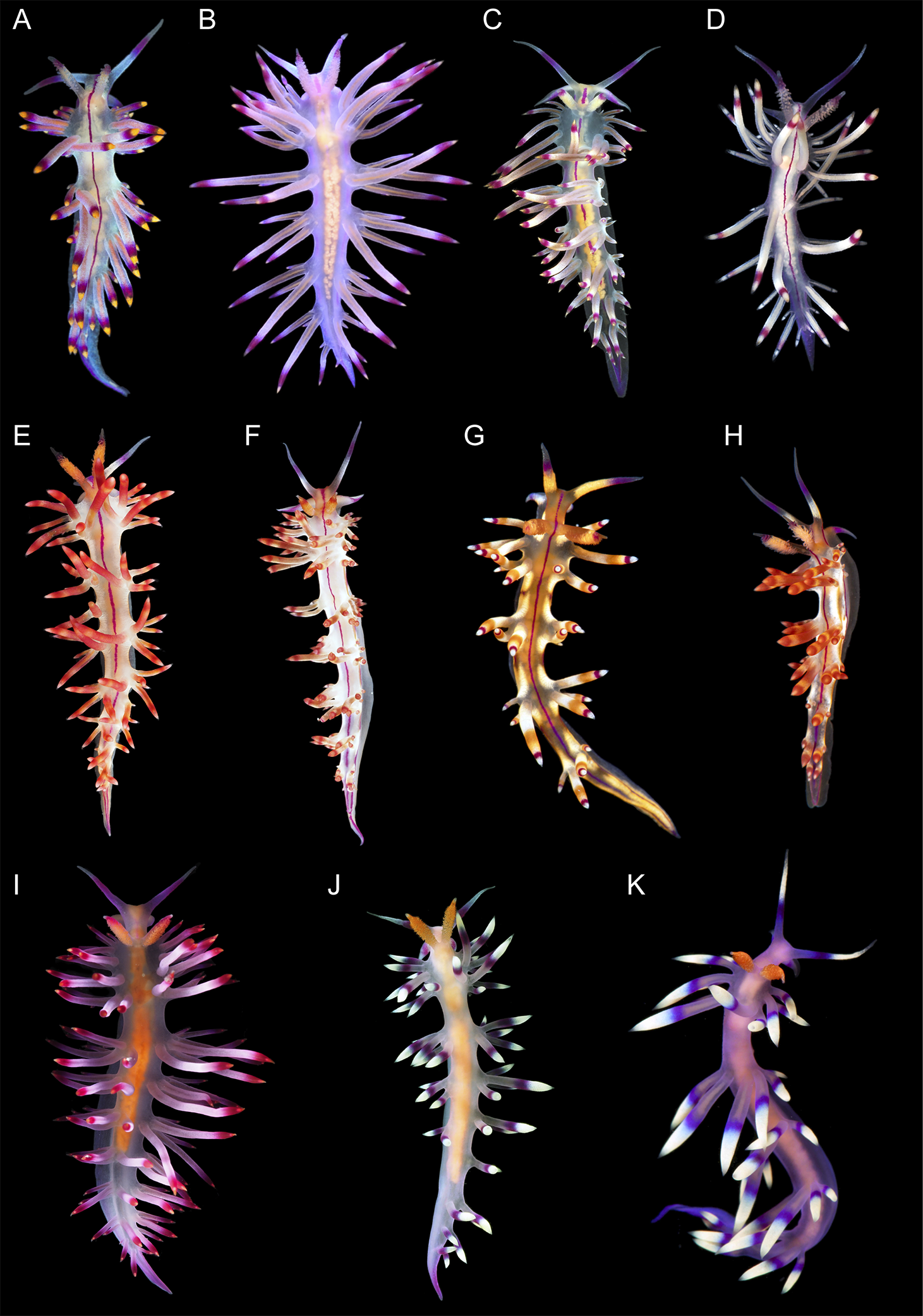

Supplement: S12 Fig — A – Coryphellina rubrolineata, voucher not specified. B – Coryphellina lotos, IZ-T9. C – Coryphellina pseudolotos N70. D – Coryphellina pannae ZIN63217. E – Coryphellina flamma ZIN63218. F – Coryphellina sp. 3 N64. G – Coryphellina sp. 2 QUP14_AC204. H – Coryphellina flamma QUP14_AC179. I – Coryphellina aurora, ZIN63224. J – Coryphellina sp. 1 N4. K – Coryphellina exoptata QUP56_AC570. Photo credits: B – Elena Mekhova, C–F, I, J –Yury Deart, Tatiana Antokhina. G, H, K – Ángel Valdés. (TIF) [file pone.0347759.s018.tif]

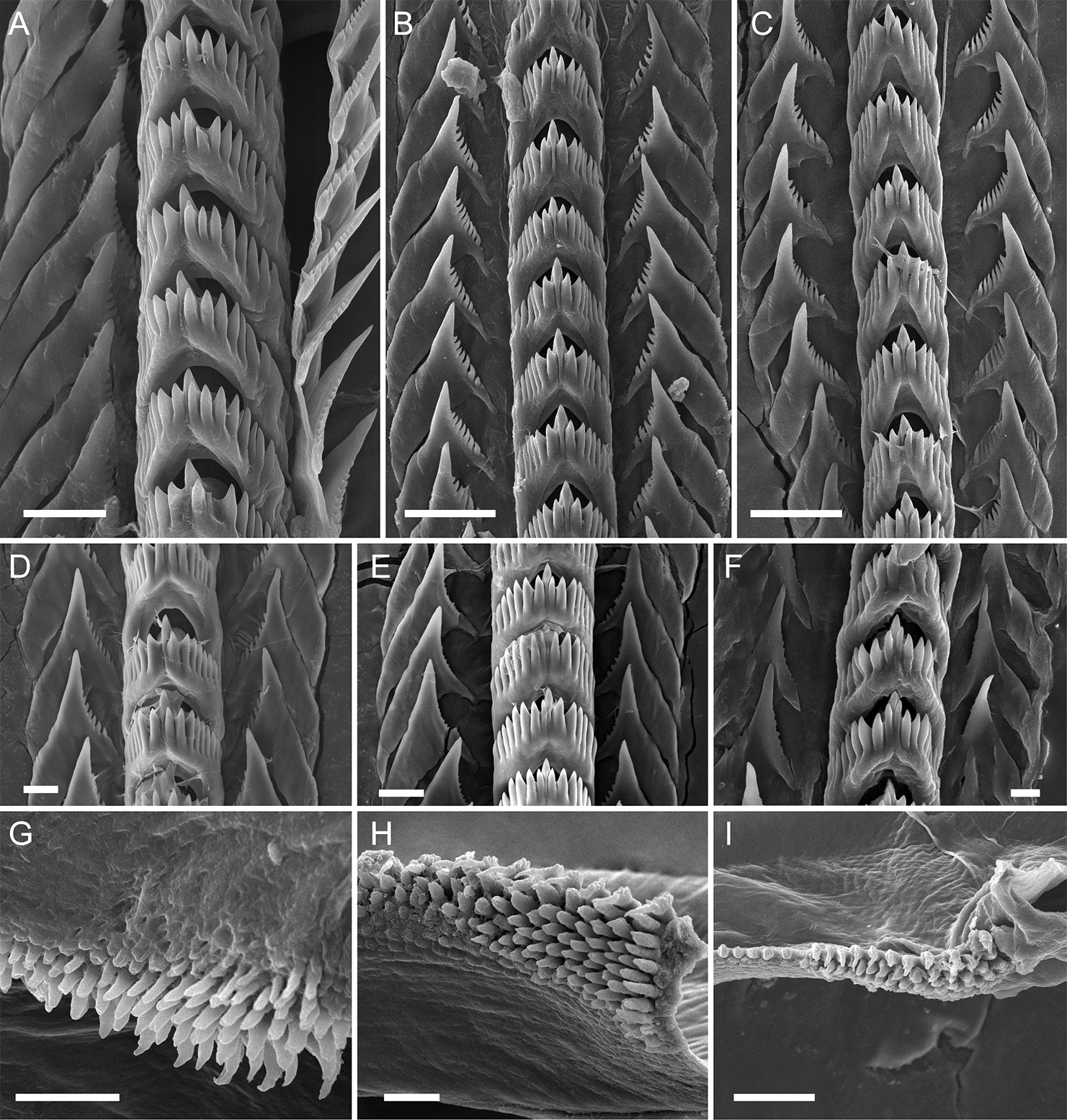

Supplement: S13 Fig — A – Coryphellina rubrolineata, IE-fr4. B – Coryphellina aurora, ZIN63224. C – Coryphellina lotos, IZ-L4. D – Coryphellina pseudolotos, N67. E – Coryphellina flamma, N63. F – Coryphellina sp. 1, L115. G – Coryphellina rubrolineata, IE-fr2. H – Coryphellina aurora, ZIN63224. I – Coryphellina flamma, ZIN63219. Scale bars: A, G = 30 µm. B, C, I = 50 µm. D, F = 10 µm. E, H = 20 µm. (TIF) [file pone.0347759.s019.tif]

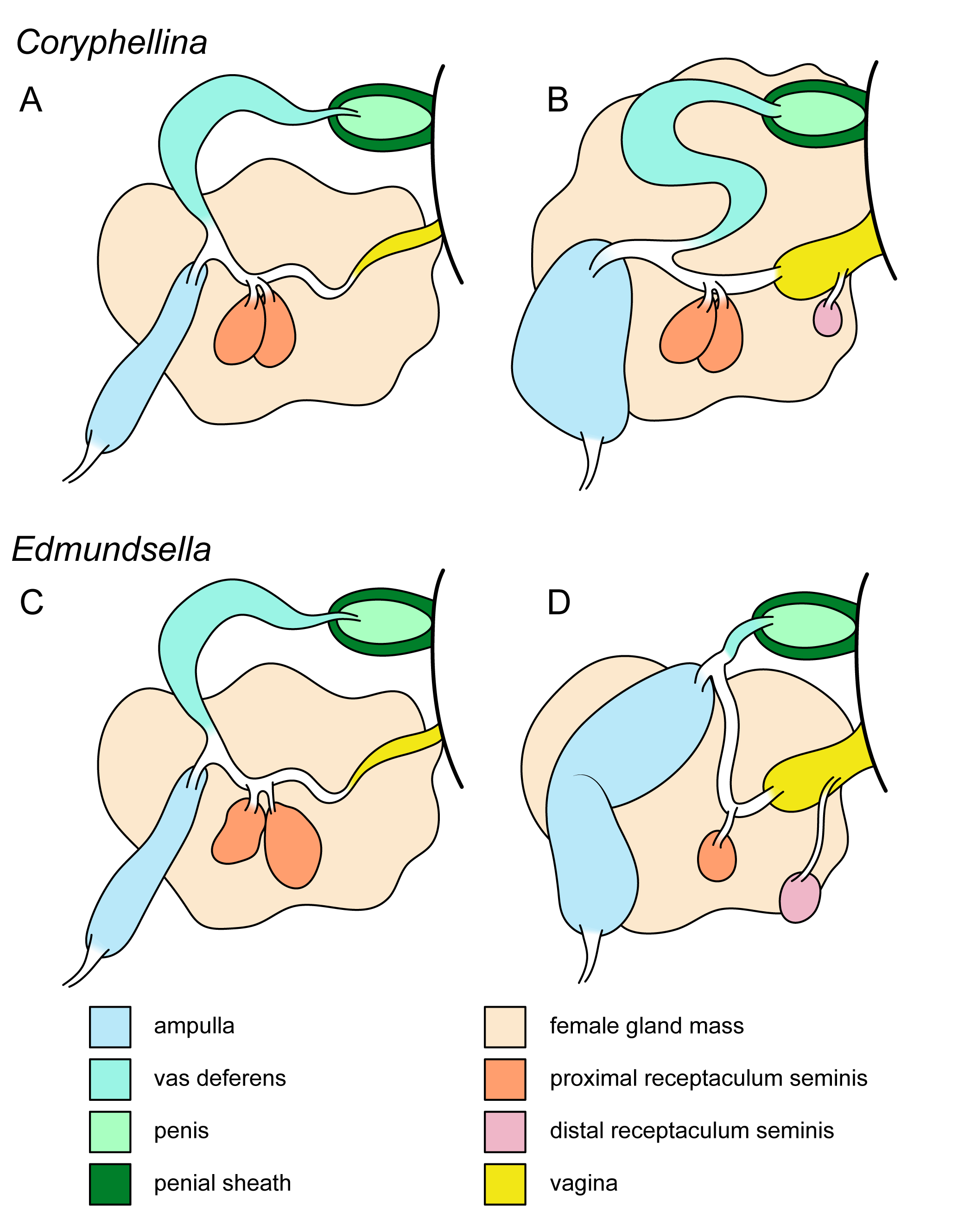

Supplement: S14 Fig — C – Coryphellina albomarginata. D – Coryphellina rubrolineata. C – Edmundsella bertschi. D – Edmundsella dushia. (TIF) [file pone.0347759.s020.tif]

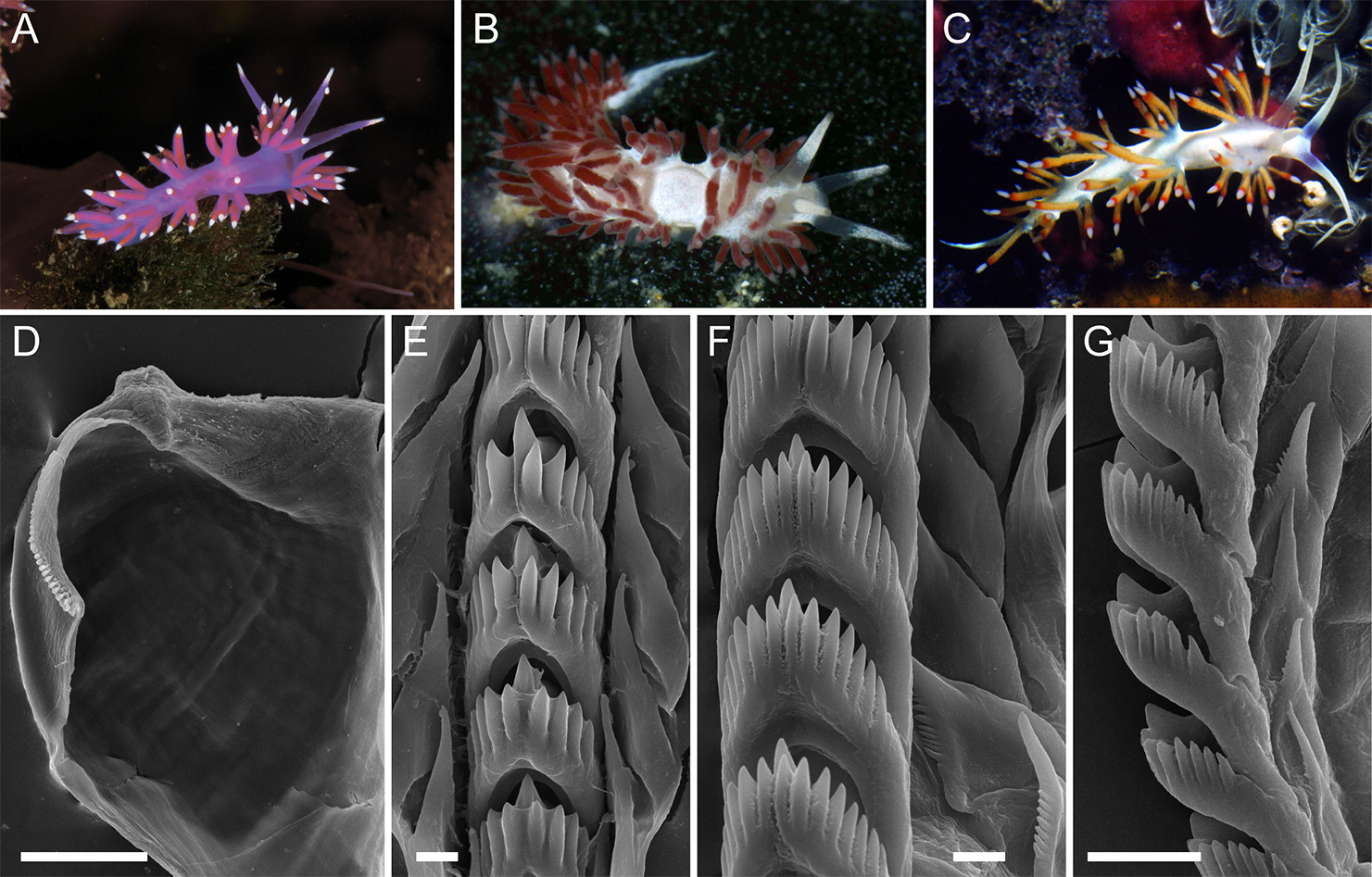

Supplement: S15 Fig — A – Edmundsella pedata, Bahia de Algeciras, voucher not specified. B – Edmundsella bertschi, CPIC1648. C – Edmundsella dushia, voucher not specified. D – Edmundsella sp., CPIC843, right jaw plate. E – Edmundsella pedata, M1, anterior radular portion. F – Edmundsella bertschi, CPIC1648, anterior radular portion. G – Edmundsella sp., CPIC843, anterior radular portion. Photo credits: A – Manuel Martínez Chacón; B, C – Ángel Valdés. Scale bars: D = 100 µm. E, F = 10 µm. G = 30 µm. (TIF) [file pone.0347759.s021.tif]

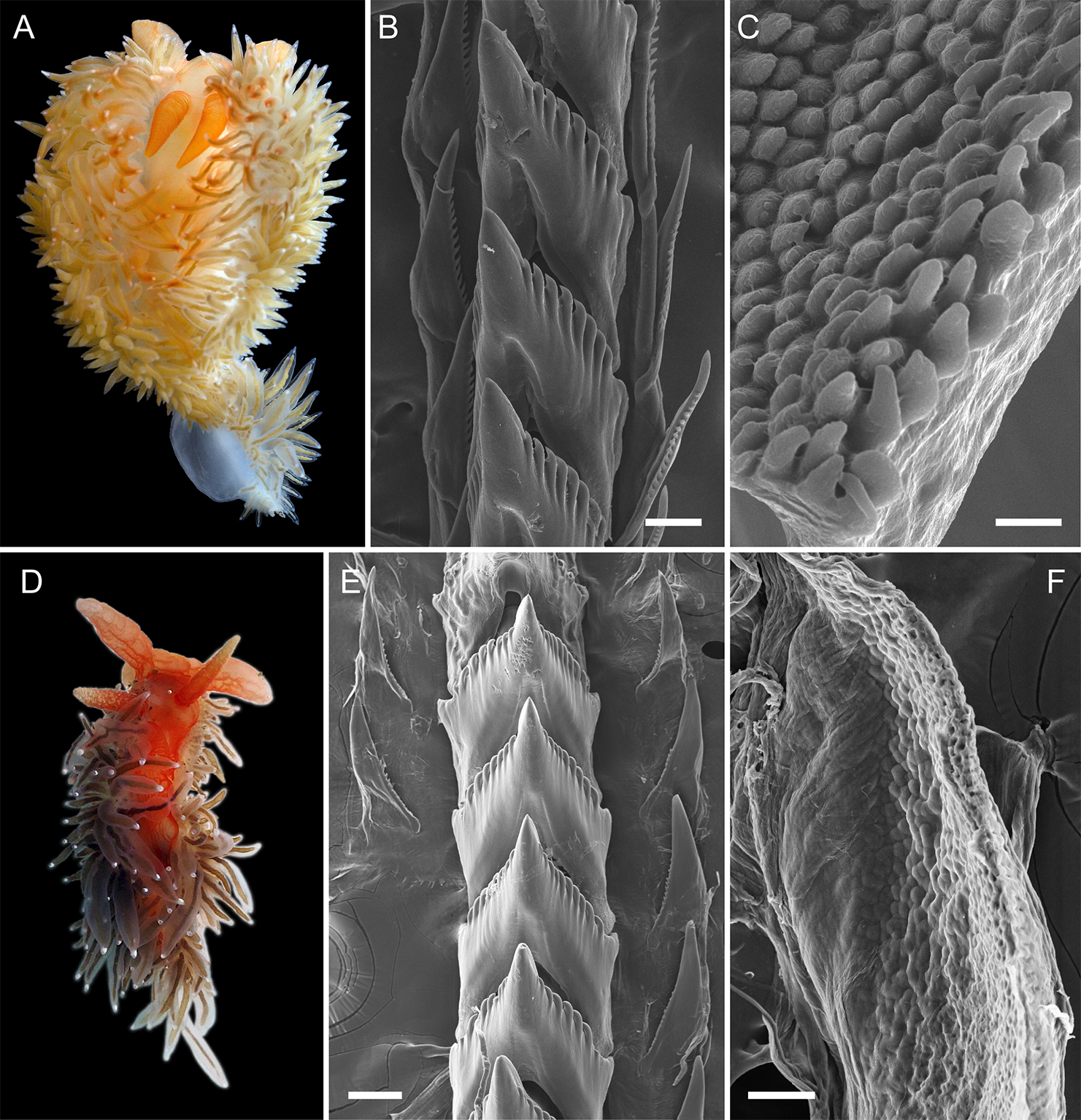

Supplement: S16 Fig — A – Ziminella vrijenhoeki, MIMB42255. B – Ziminella vrijenhoeki, MIMB42255, radula. C – Ziminella vrijenhoeki, MIMB42255, masticatory border of jaw. D – Ziminella japonica, MIMB50760. E – Ziminella japonica, MIMB50759−1, radula. F – Ziminella japonica, MIMB50759−1, masticatory border of jaw. Photo credits: A – Anastasia Maiorova; D – Olga Chichvarkhina. Scale bars: D–F, H = 20 µm. G = 200 µm. (TIF) [file pone.0347759.s022.tif]

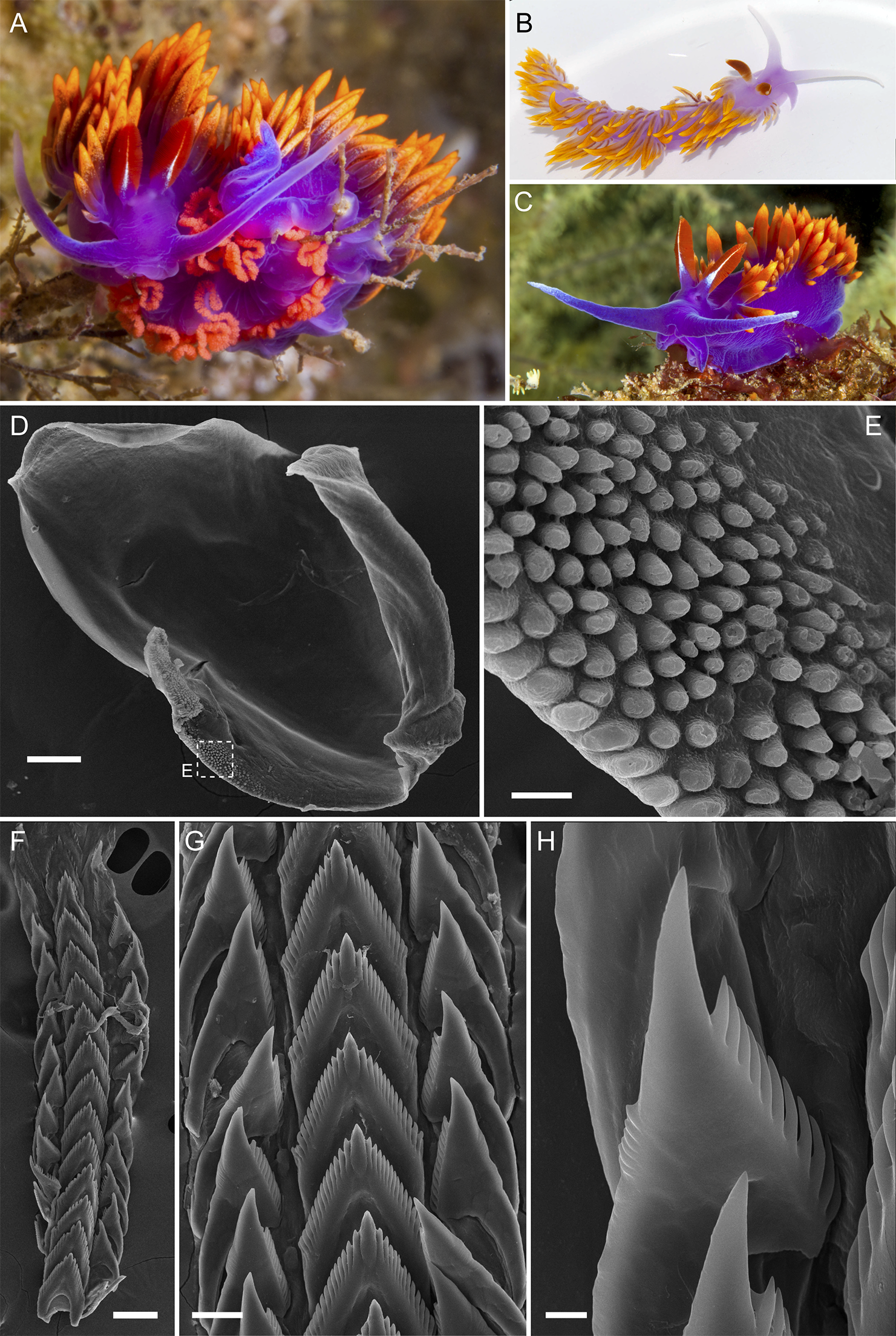

Supplement: S17 Fig — A – voucher not specified, specimen from Palos Verdes, Southern California, USA. B – CPIC1015. C – voucher not specified, specimen from Bahía de los Ángeles, Baja California, México. D – CPIC1459, left jaw plate. E – CPIC1459, details of masticatory border denticulation. F – CPIC1459, median and posterior radular portions. G – CPIC1033, rachidian and lateral teeth, median radular portion. H – CPIC1459, lateral tooth, note denticulation of outer surface. Scale bars: D = 100 µm. F, E, H = 10 µm. G = 50 µm. (TIF) [file pone.0347759.s023.tif]

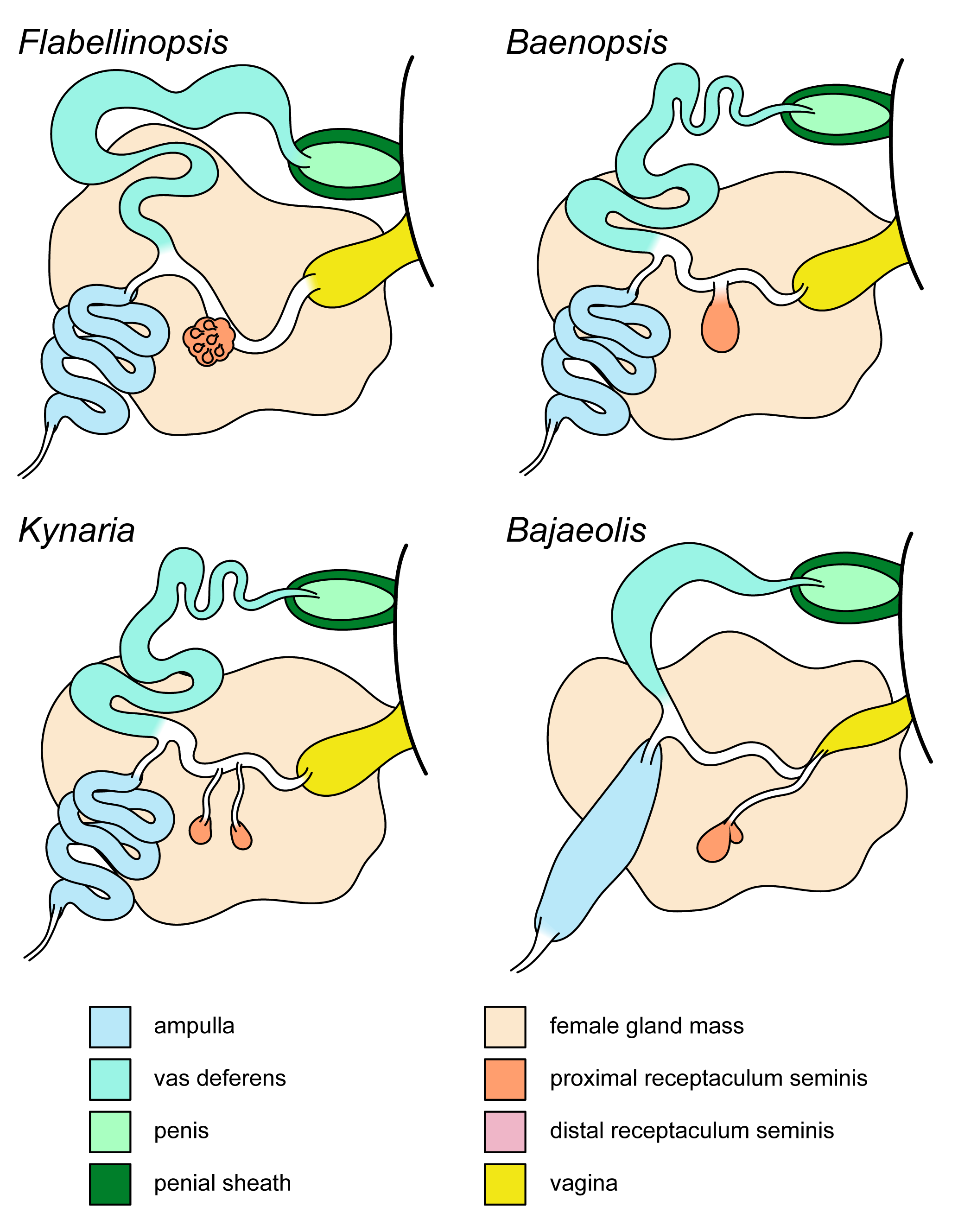

Supplement: S18 Fig — (TIF) [file pone.0347759.s024.tif]

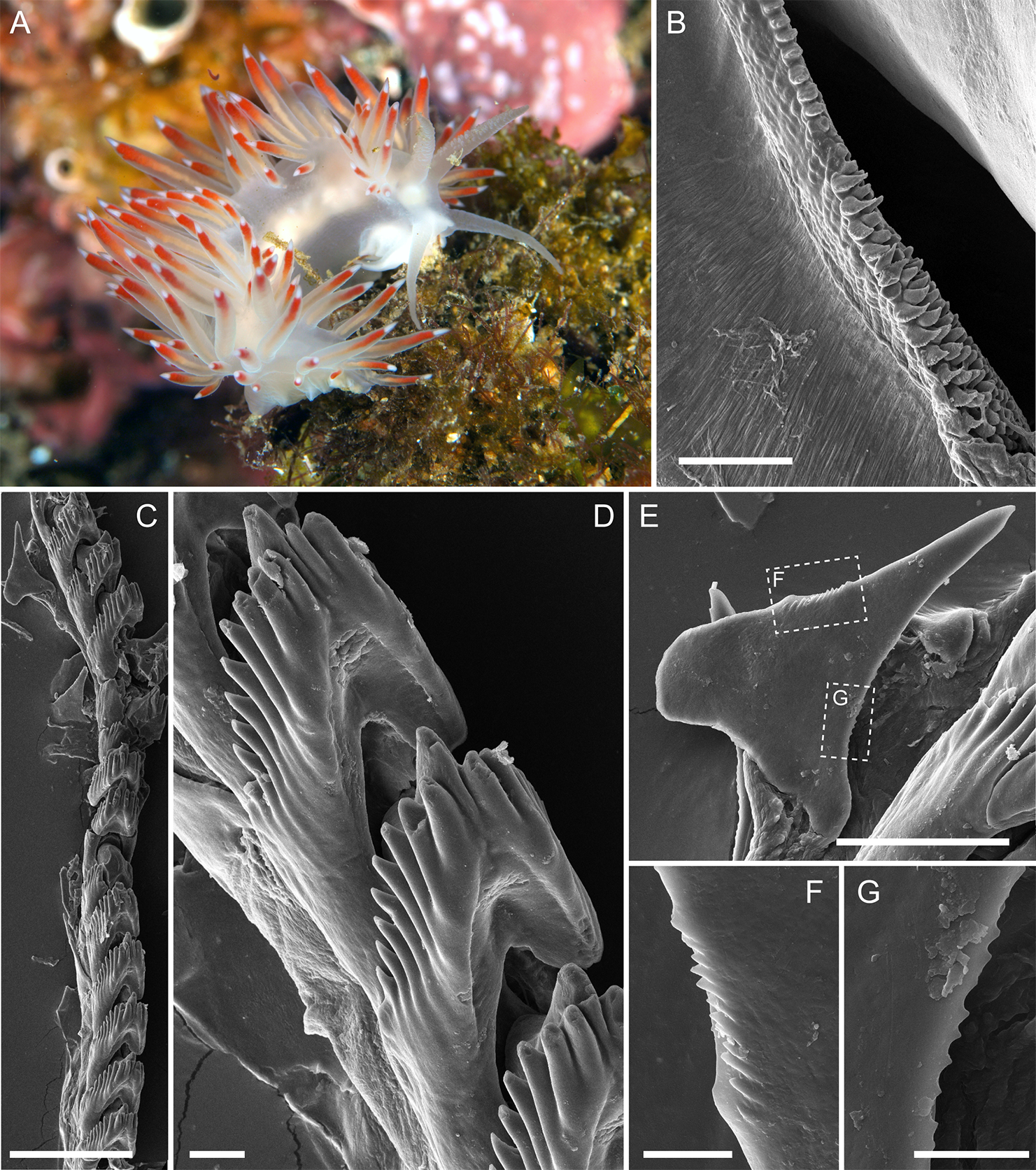

Supplement: S19 Fig — A – living photo, specimen was not collected. B–G – MNCN15.05/98989, buccal armature (SEM). B – masticatory border of jaw. C – radula. D – rachidian teeth. E – lateral tooth. F, G – details of lateral teeth outer (F) and inner (G) denticulation. Scale bars: B = 50 µm. C = 100 µm. D = 10 µm. E = 30 µm. F, G = 5 µm. (TIF) [file pone.0347759.s025.tif]

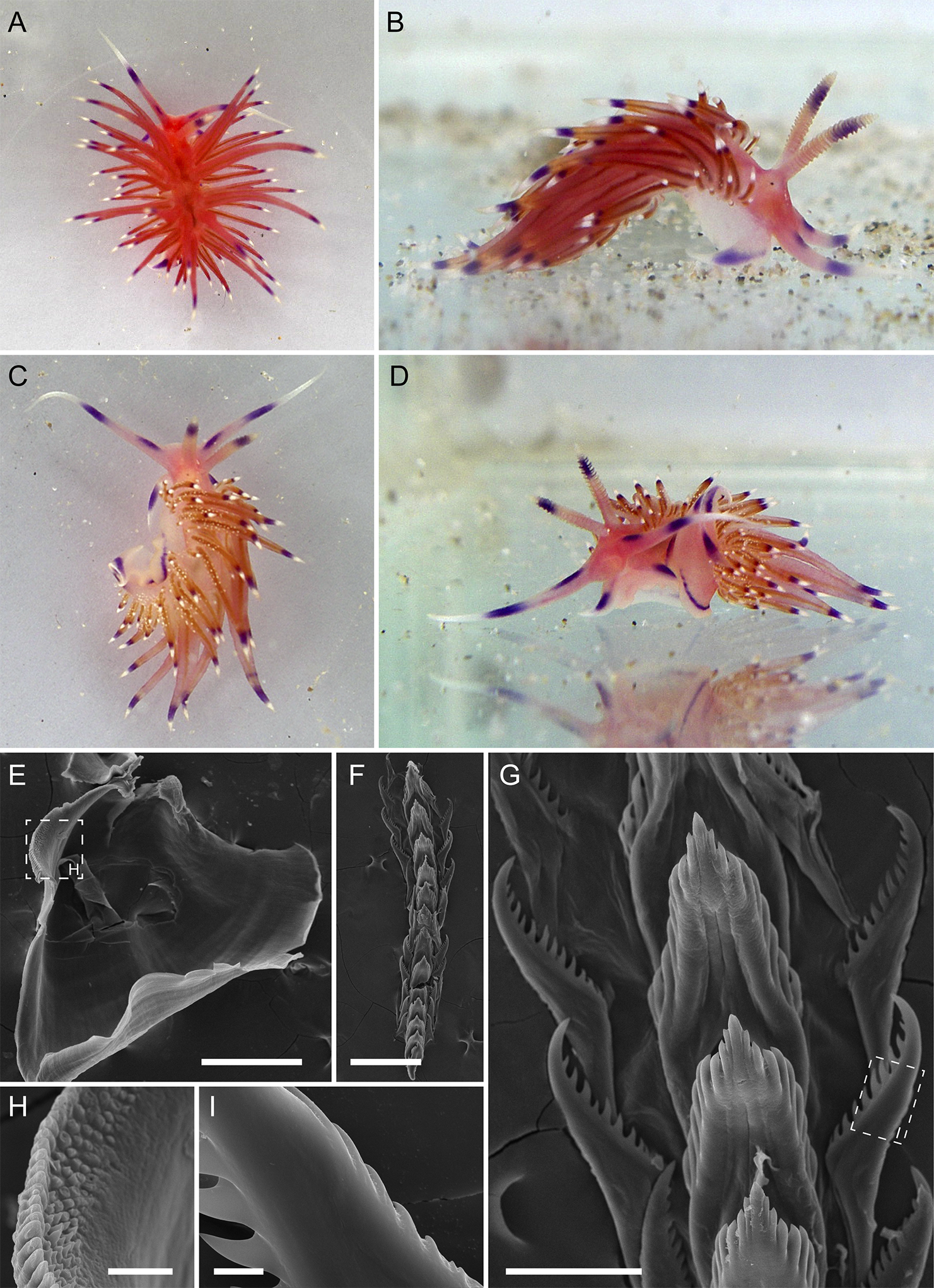

Supplement: S20 Fig — A – CPIC579. B–D – CPIC581. E – right jaw plate, CPIC579. F – radula, CPIC579. G – rachidian and lateral teeth, CPIC579. H – details of denticulation of masticatory process. I – details of denticulation of lateral teeth outer surface. Scale bars: E, F = 300 µm. G = 100 µm. H = 30 µm. I = 10 µm. (TIF) [file pone.0347759.s026.tif]

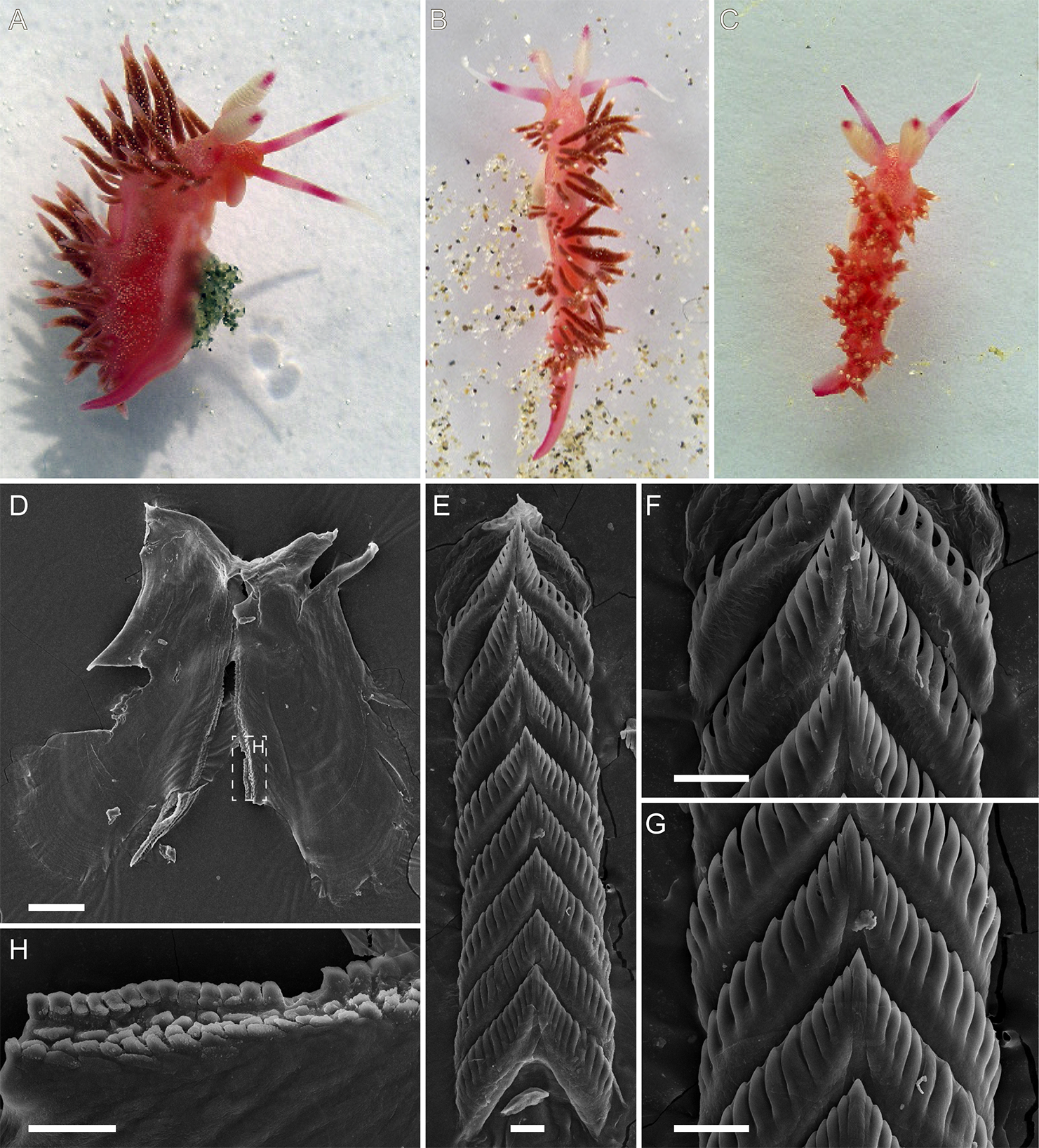

Supplement: S21 Fig — A – CPIC0597. B – CPIC0582. C – CPIC0592. D – paired jaw plates, CPIC592. E – radula, CPIC592. F – rachidian teeth, posterior radular portion, CPIC592. G – rachidian teeth, middle radular portion, CPIC592. H – details of denticulation of jaws masticatory border. Scale bars: D = 100 µm. E–H = 20 µm. (TIF) [file pone.0347759.s027.tif]

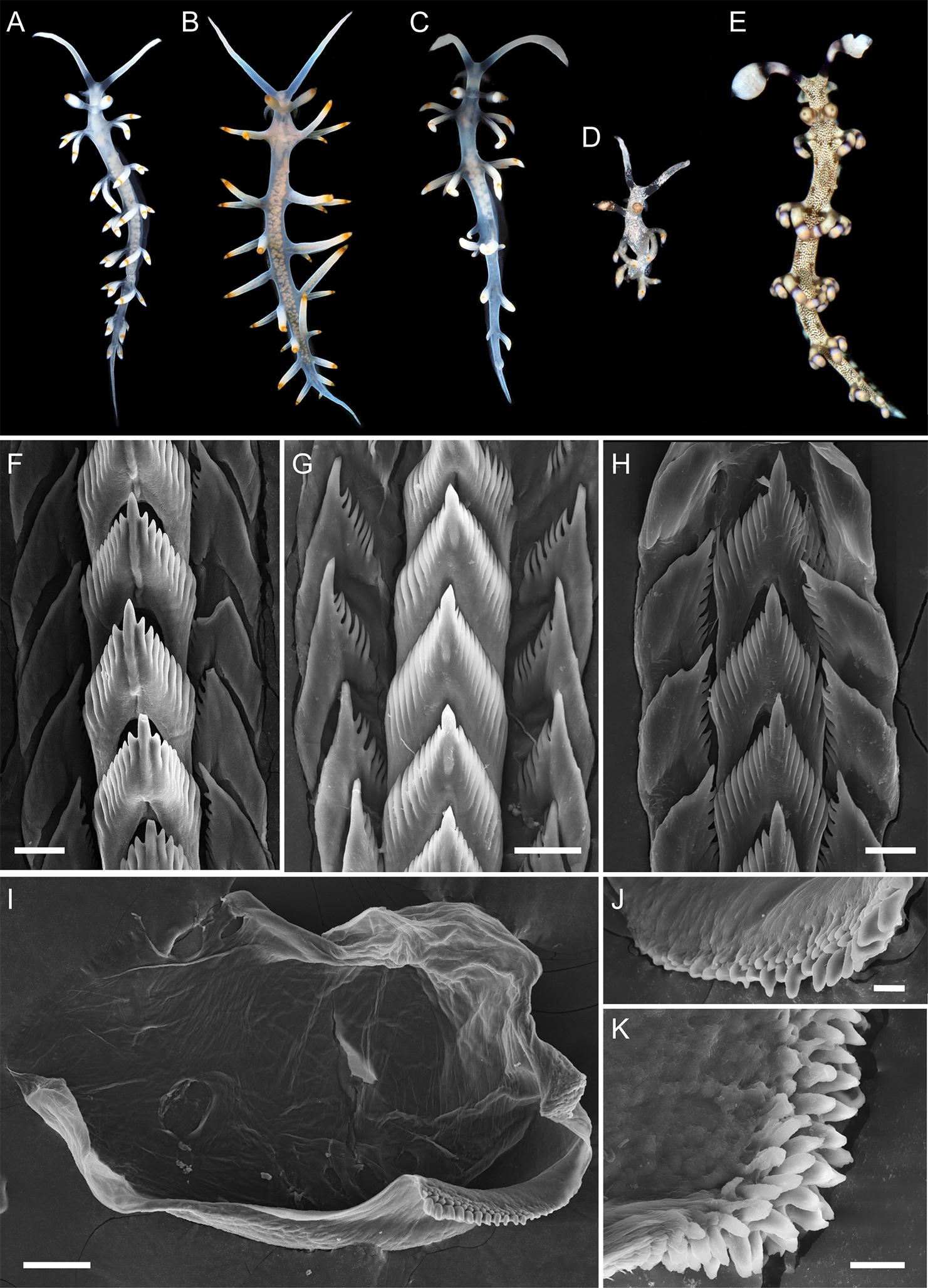

Supplement: S22 Fig — A – Samla bicolor, n19. B – Samla takashigei, n378. C – Samla sp. 1, n20. D – Samla sp. 2, n325. E – Samla riwo, QNAL-KA-GF005. F – Samla takashigei, n378, radula. G – Samla bicolor, n52, radula. H – Samla riwo, QNAL-HA-JD346, radula. I – Samla bicolor, n52, jaw. J – Samla bicolor, n52, masticatory border of jaw. K – Samla takashigei, n378, masticatory border of jaw. Photo credits: A–D – Yury Deart, Tatiana Antokhina. F – Angel Valdes. Scale bars: F–H = 20 µm. I = 200 µm. J, K = 10 µm. (TIF) [file pone.0347759.s028.tif]

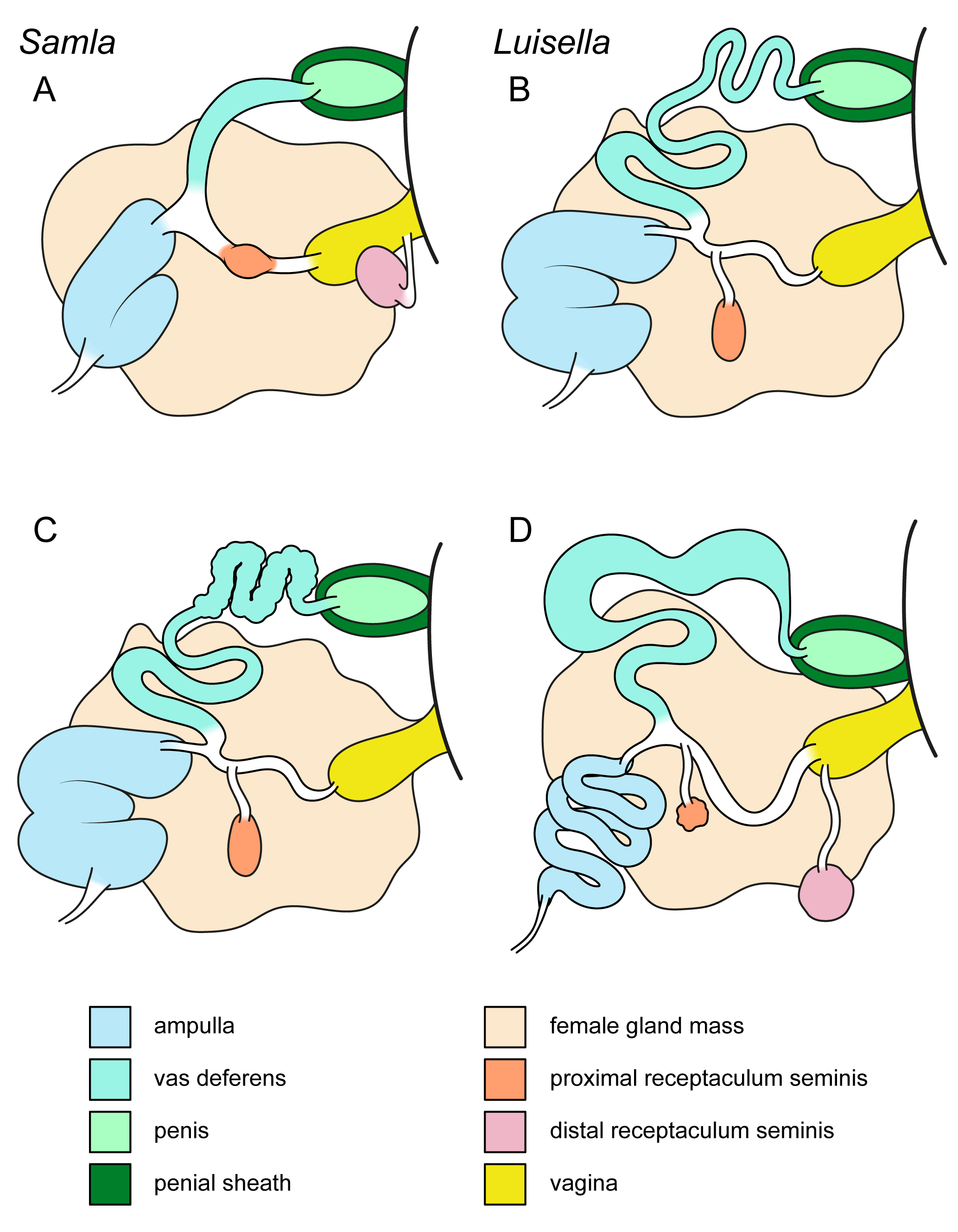

Supplement: S23 Fig — A – Samla bicolor. B – Luisella engeli. C – Luisella babai. D – Luisella telja. (TIF) [file pone.0347759.s029.tif]

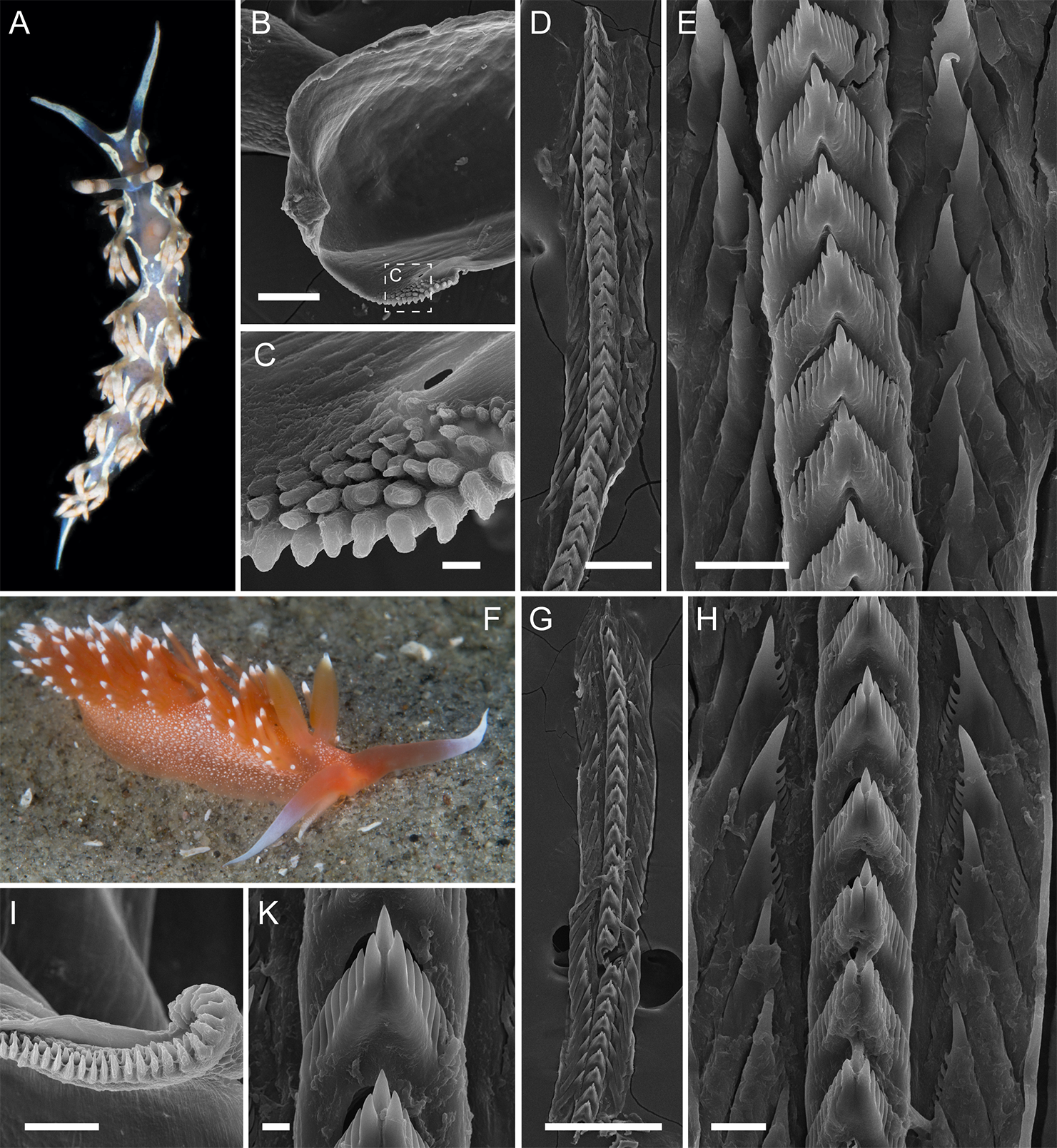

Supplement: S24 Fig — A–E – Luisella engeli, CPIC1582. F–H – Luisella telja, CPIC2110. A – living specimen. B – anterior part of jaw. C – masticatory process of jaw. D – radula. E – posterior radular portion. F – living specimen. G – radula. H – posterior radular portion. I – masticatory process of jaw. K – rachidian tooth. Scale bars: B, D = 100 µm. C, K = 10 µm. E, H, I = 30 µm. G = 300 µm. (TIF) [file pone.0347759.s030.tif]

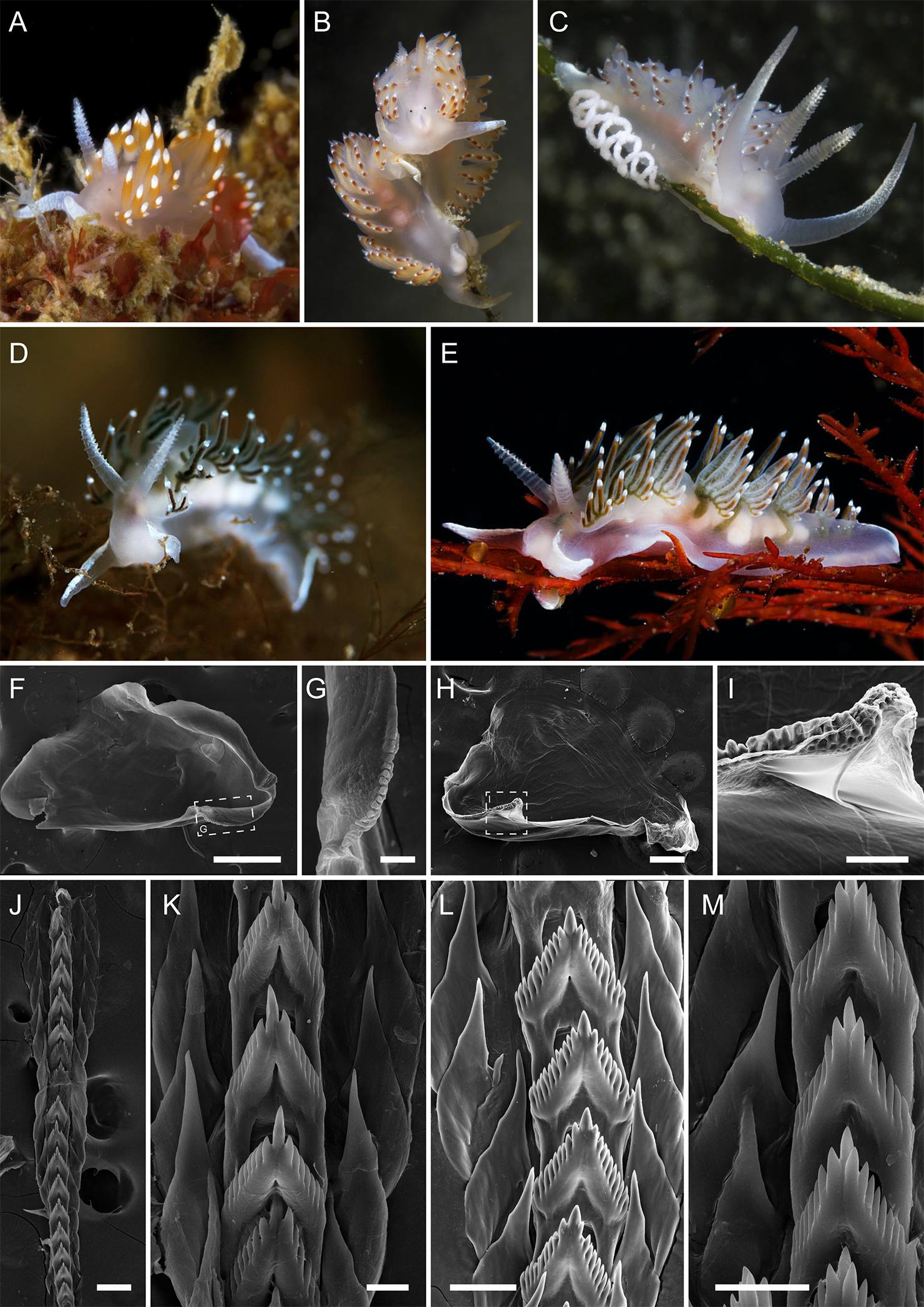

Supplement: S25 Fig — A – Apata cf. pricei, from Bahía de los Ángeles, Mexico, not collected. B, C – Apata cf. pricei, from San Diego, California, not collected. D, E – Apata pricei kommandorica, voucher not specified. F, G, J, K – Rud21177. H, I, L – Rud21284. M – CPIC1020. F, H – jaw. G, I – masticatory process. J–M – radula. A – living specimen, voucher not specified. B – anterior part of jaw. C – masticatory process of jaw. D – radula. E – posterior radular portion. F – living specimen. G – radula. H – posterior radular portion. I – masticatory process of jaw. K – rachidian tooth. Photo credits: A–C: Craig Hoover. D, E: Andrey Shpatak. Scale bars: F = 300 µm. G, K, M = 30 µm. H, J = 100 µm. I, L = 50 µm. (TIF) [file pone.0347759.s031.tif]

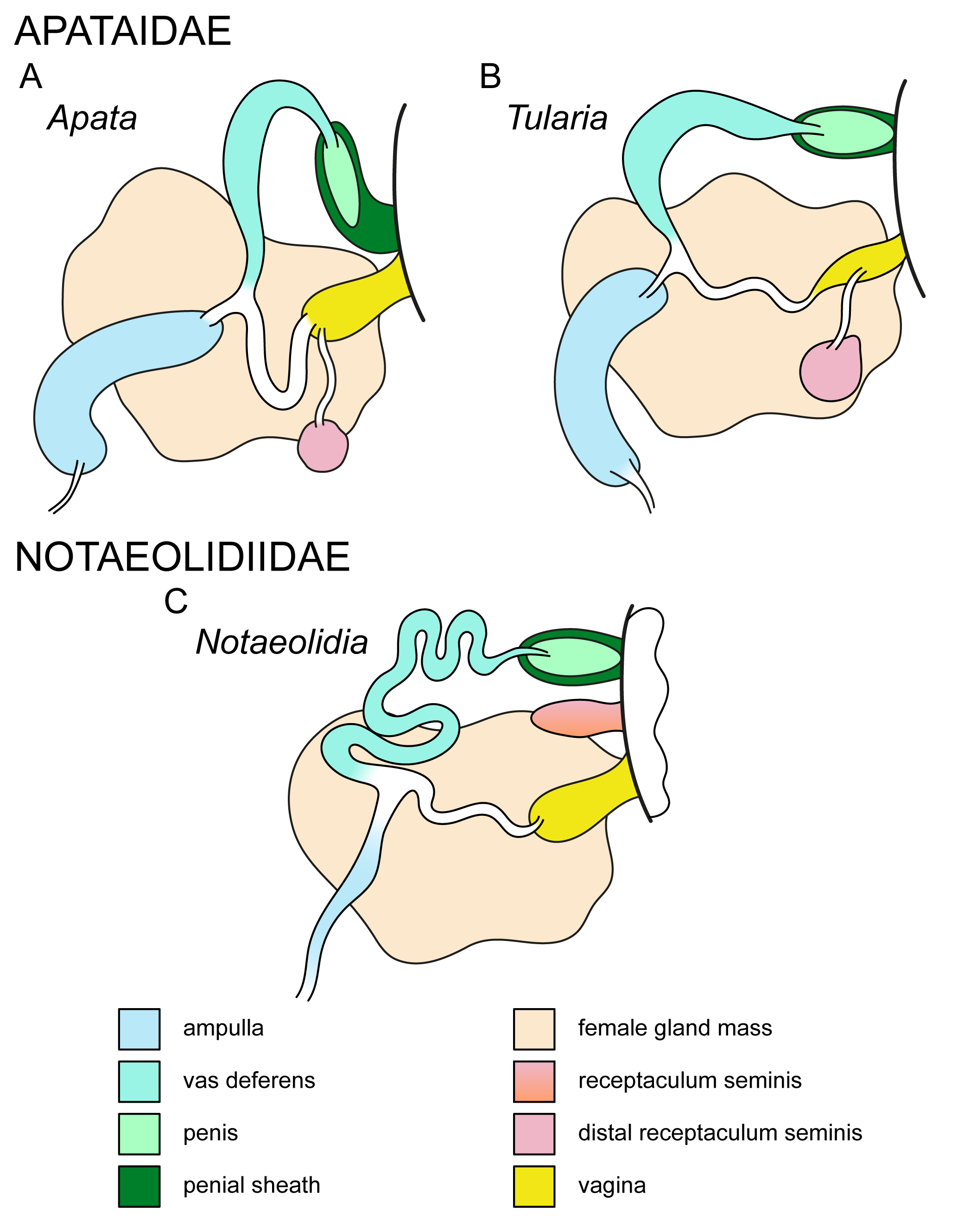

Supplement: S26 Fig — (TIF) [file pone.0347759.s032.tif]

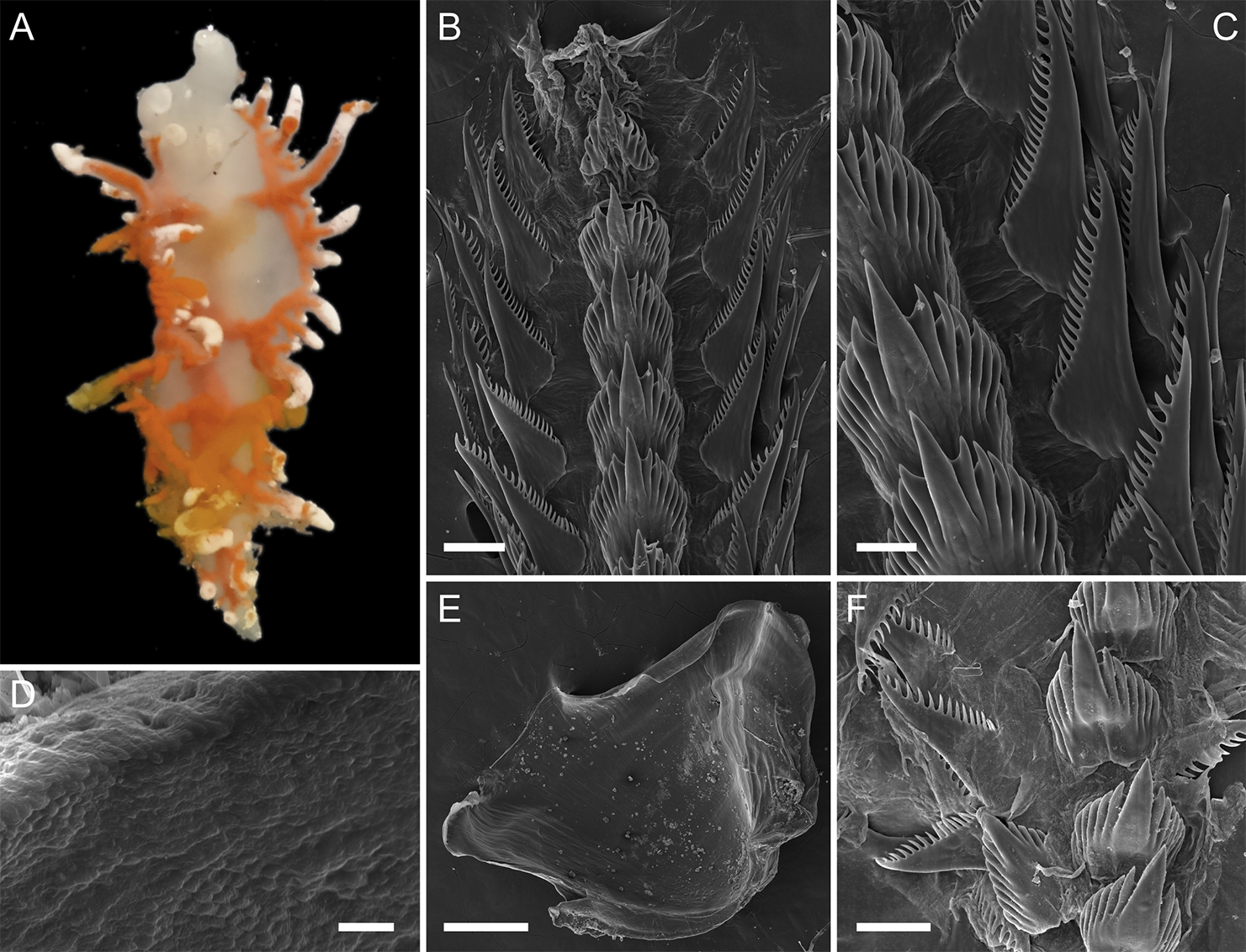

Supplement: S27 Fig — A – Notaeolidia cf. depressa, Nd1. B–E – Notaeolidia depressa, ZIN1. B, C – radula. D, E – jaws. F – Notaeolidia cf. depressa, Nd1, radula. Photo credit: A – Olga Bozhenova. Scale bars: B = 100 µm. C, F = 50 µm. D = 10 µm. E = 500 µm. (TIF) [file pone.0347759.s033.tif]

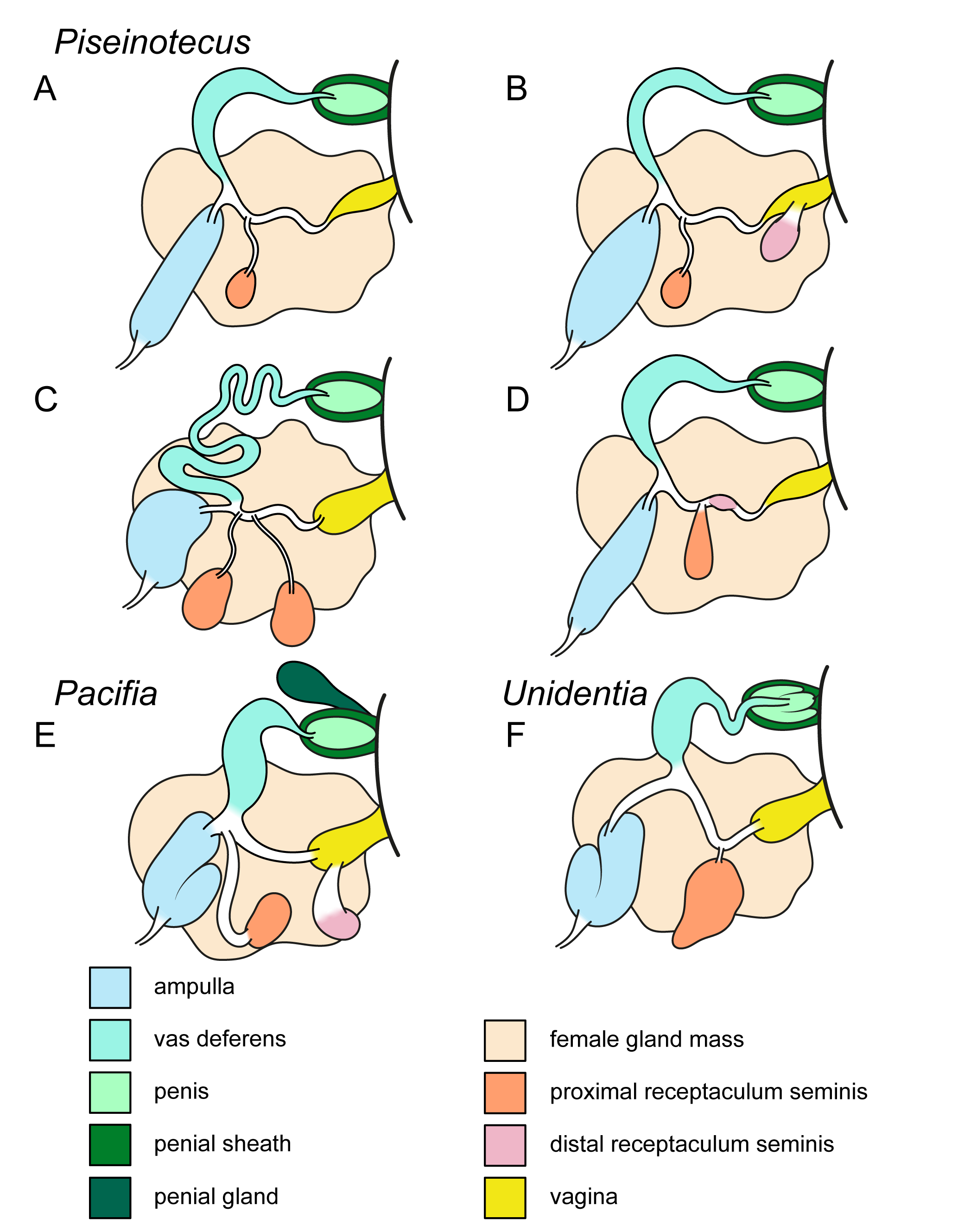

Supplement: S28 Fig — A – Piseinotecus divae. B – Piseinotecus soussi. C – Piseinotecus minipapilla. D – Piseinotecus gonja. E – Unidentia angelvaldesi. F – Pacifia goddardi. (TIF) [file pone.0347759.s034.tif]

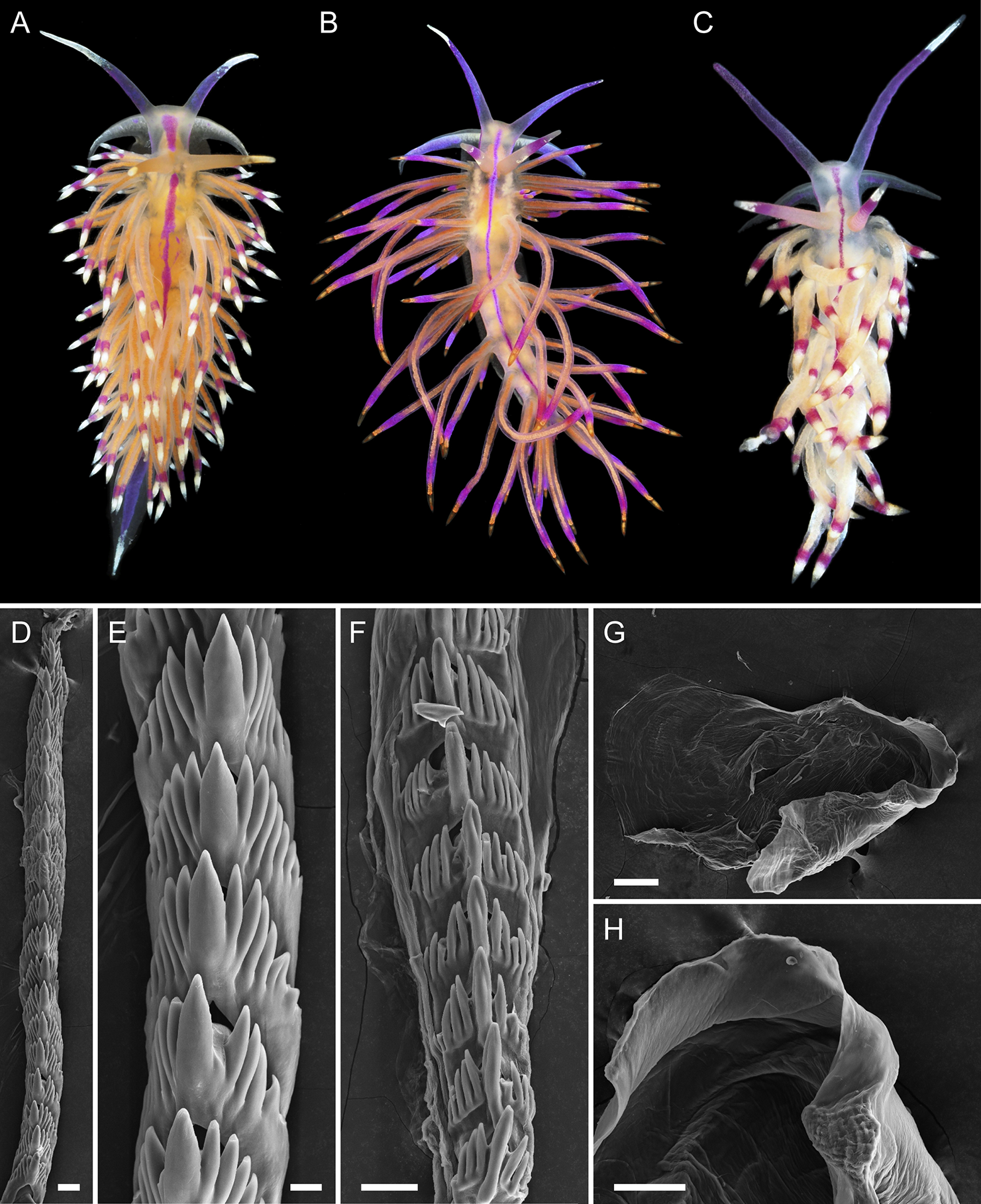

Supplement: S29 Fig — A – Unidentia aliciae, N101. B – Unidentia sandramillenae, N432. C – Unidentia sp. 1, N313. D, E – Unidentia aliciae, N102, radula. F – Unidentia sandramillenae, N432, radula. G, H – Unidentia aliciae, N101, jaws. Photo credits: A–C – Yury Deart, Tatiana Antokhina. Scale bars: D, F = 30 µm. E = 10 µm. G = 100 µm. H = 50 µm. (TIF) [file pone.0347759.s035.tif]

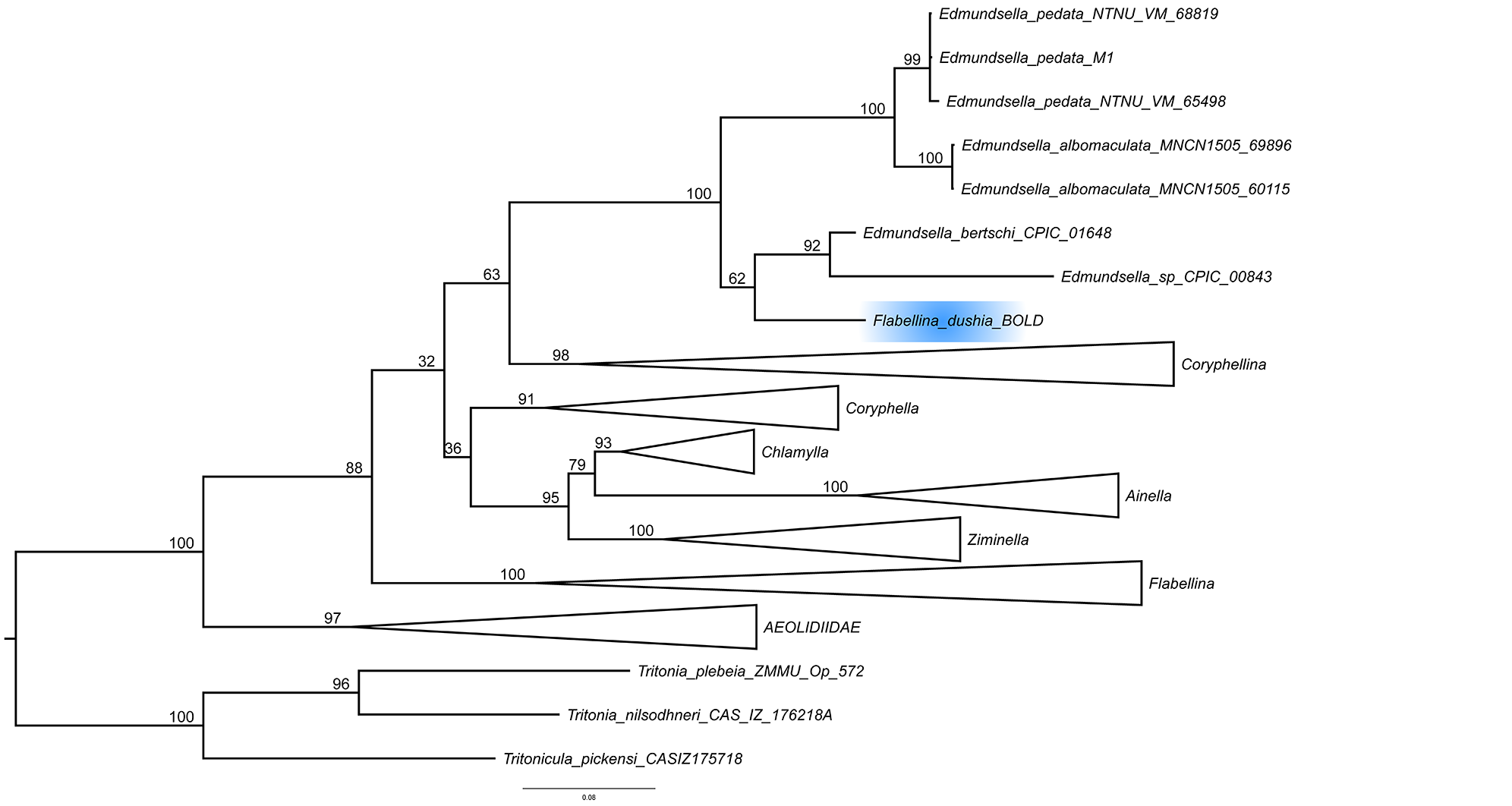

Supplement: S30 Fig — Numbers on nodes indicate bootstrap support from ML. (TIF) [file pone.0347759.s036.tif]
