# Supplementary material for: Neither “lumpers” nor “splitters”: A global revision of Flabellinidae s.l. nudibranchs (Gastropoda: Heterobranchia: Nudibranchia)
Source: PLoS One. 2026 May 20;21(5):e0347759. doi: 10.1371/journal.pone.0347759 (PMC13189321; doi:10.1371/journal.pone.0347759)
Supplement: Attachment — Submitted filename: PONE-D-25-43242_comments.pdf [file pone.0347759.s037.pdf]

**General comments**

The authors present a complex and detailed study on the systematics of the family Flabellinidae, a group whose taxonomic status has long been problematic due to both historical and recent conflicting classifications. The manuscript provides a useful and comprehensive overview of the history of classification issues in the family which serves as a solid framework for the study. The amount of data analyzed and the methodology employed are appropriate, reflecting meticulous work and considerable effort that deserves acknowledgment. The integration of phylogenetic analyses with morphological traits is a major strength of the manuscript.

The paper is generally well written and logically structured. That said, the text would benefit from a light language revision to shorten some of the longer sentences and to reduce repetition, making it more concise and easier to follow. This would improve readability and enhance the impact of the results on the reader.

Overall, the authors demonstrate strong command of methods and background knowledge. The study represents a valuable contribution that advances our current understanding of flabellinid systematics. The high-quality figures and graphics further support the work. This is clearly the result of substantial effort, which should be recognized and valued. I recommend the manuscript for publication after minor revisions.

**Abstract**

General comments: While the abstract is clear and it successfully conveys the objectives and rationale of the study it is a bit too long and it would benefit from some tightening and rephrasing to improve clarity and impact. The key results and taxonomic proposals are important, but too much detail is included, please consider summarizing the new taxa and emphasizing the broader systematic implications. A short abstract is always "powerful".

Line 29-30: The sentence beginning with "it is extremely challenging…" could be separated into two sentences.

Line 31: The phrase "in its traditional sense" is vague. I also believe that the sentence could be rephrased to avoid repetition (e.g.: "Recent studies have shown that the group is polyphyletic and have suggested reclassifying the 84 described species into 29 genera across 7 families.")

Line 33: I´d eliminate "on the taxonomy and phylogeny", as this is already implied.

Line 34: Replace "and/or" with just "and".

Line 44: Typo: “results shown” should be “results showed” or “results show”, depending on whether the authors want to keep the abstract in past or present tense.

Line 52: Replace “most likely represents“ with just “likely represents”.

**Introduction**

General comments: A useful and comprehensive overview of the long history of classification issues in Flabellinidae s.l. is provided. However, it is a bit overly detailed, which risks losing the reader. I recommend reducing background information that has already been summarized in earlier reviews (e.g., [22]) and focusing more directly on the research gap to justify the present study. Some sentences are quite long and can be modified to improve readability.

Line 58: Rephrase to (…) conflict between ‘lumpers’ and ‘splitters [1]. This issue, mainly driven by reliance on ambiguous morphological traits… (change position ambiguous to improve gramma).

Line 59–60: Simplify to “…resulted in both over- and underestimations of diversity. The existence of cryptic diversity further exacerbated the underestimation problem…”

Line 65: “suggesting and end” instead of “evoking the end” is in my opinion a better choice of words.

Line 68: I recommend adding “can” so the sentence reads: “where the lack of clear definitions can make taxonomic decisions on genera and families highly subjective.” This small change emphasizes that subjectivity is a potential issue rather than a common situation. In my opinion, this nuance is important, as taxonomy is not inherently subjective when supported by thorough and well-structured work.

Line 69–71: Break into two sentences (as my suggestion in the abstract) “…cryptic diversity, plastic ecology, and variable COI divergence across groups make morphological interpretation extremely challenging. As a result, it is difficult to reach a consensus…”

Line 73: I suggest simplifying the sentence (e.g.: Flabellinidae s.l. is a large cosmopolitan group of nudibranchs, with more than 90 described species [8])

Line 75–77: This sentence can be break after “two ‘main’ genera”

Line 81–83: “in contrast” can be replaced by “vs” to simplify

Line 111–113: Simplify to “…Most of these genera” instead of “Most of the genus-level taxa”

**Material and Methods**

General comments: This section is detailed and covers all the needed information. However, some sentences can be modified a bit to make them more concise or grammatically precise.

Line 132: Rephrase: "For this study, we used specimens from extensive museum collections deposited at..."

Lines 133–136: The list of institutions can be a bit clearer by adding some information between brackets: e.g., California State Polytechnic University Invertebrate Collection (CPIC, Pomona, USA), (MNHN, Paris, France)

Line 138: Modify to "between 2011 and 2024."

Line 143: It should be "...are described in detail in [34–36]." (detail without the final S)

Line 144: The use of dashes makes the reading a bit difficult, instead you could write it more smoothly ""...described in detail in [34–36]; those used at California State Polytechnic University are described in [37]; and those at the Instituto Universitario de Investigación Marina (INMAR), University of Cádiz, in [38]."

Line 153–154: This sentence can be reduced (e.g.:"Previously published datasets [22, 28, 34, 40] were incorporated, with Tritoniidae selected as a distant outgroup along with representatives of all major families of Aeolidoidea.")

Line 171: Consider a more neutral sentence: "...visualized in FigTree 1.4.0 and finalized in Adobe Illustrator CS 2015."

Line 175.177: Although the meaning of the sentence is clar, I believe it can be improved for better readability (e.g.: Since our goal is to clarify the taxonomy of Flabellinidae s.l. at the genus and family level, species diversity and identity were not the focus, and no species delimitation analyses were performed.")

Line 178–179: Very long; suggest breaking into two sentences for readability.

Line 183: Consider being more concise: "...examined for external morphology under a stereomicroscope."

Line 193–194: Could be shortened to: "...dissected dorsally along the midline and examined under a stereomicroscope."

**Results – Molecular Phylogeny**

General comments: Without dismissing the quality of the research (that is of high standards) the section is overall, very dense and can overwhelm the reader.

- Please, consider summarizing some repetitive descriptions (e.g., node supports, repeated morphological states) and referring readers to supplementary tables/figures. (e.g., lines 212 to 225)

- The text describes complex clades in a single long sentence in several parts of the text. Breaking these into shorter sentences would improve readability.
- Figures are clear, but cross-referencing between text and figures could be more direct (e.g., "see Fig. 1A" rather than just "see Fig. 1") and included in more parts of the results.
- Please ensure consistent use of family and genus names (e.g., Flabellinidae s.str. vs. Flabellinidae sensu stricto). In some places, terms like "recovered" and "clustered" could be simplified to avoid repetition.
- The morphological analysis. Consider highlighting which traits were most informative for higher-rank taxonomy, and which ones were too variable to be useful.
- Some detailed species-level results (e.g., multiple clades of *Samla bicolor*) could be shortened. This would keep the focus on the main findings relevant to higher-level taxonomy.

Line 197: "from 130" instead of "for"

Line 199: Change the position of latter: "the latter two partitions were trimmed with Gblocks."

Line 203–204: The use of "compromise" sounds weird, consider changing "…and of the genera Edmundsella Korshunova et al., 2017 and Samla was compromised…" for something as "…and the genera Edmundsella and Samla were not recovered as monophyletic…"

Line 217: "…split into two distinct sister clades…" is redundant, I´d say "…split into two distinct clades…" instead.

Line 222–224: Modify: "unexpectedly grouped in a monophyletic clade…"

Line 236–238: Consider replacing "…which conforms previously recovered relationships…" by "…which are consistent with previously recovered relationships…"

Line 240: Can be simplify as "The ML tree suggested a different topology among subclades, but without support."

Line 246: Correct as "…was recovered as sister to Coryphella…"

Line 247: "Add comma: "Within Flabellinidae s.str., three large clades were recovered."

Line 265–269: These sentences are a bit difficult to follow. Please rephrase.

Line 282–284: Consider rephrasing, e.g "…but this relationship lacked support in both BI (PP = 0.74) and ML (BS = 48)."

Line 291–293: Add comma: "With the exclusion of S. rubropurpurata and S. telja, the genus Samla was recovered as monophyletic…"

Line 299–301: Simplify "...including Piseinotecus soussi..."

Line 305–306: Modify "...instead it formed a polytomy with the other P. goddardi specimens and P. amica..."

Line 313–315: I´d be more cautious with the sentence maybe something like "...suggesting they may represent two distinct species."

**Results – Systematics**

The *Systematics* section is well structured and supported by valuable details. It shows clear expertise, and the supplementary tables compiling all morphological characters are particularly valuable for future research. The descriptions are generally clear and informative. However, several family- and genus-level diagnoses are too broad, relying mainly on variable traits. Refining these diagnoses to emphasize consistent or unique character combinations would make them more useful and would provide stronger morphological support to complement the molecular justification for the new genera.

**Discussion**

General comments: As in previous sections, the discussion is through and demonstrates strong knowledge. This section effectively places the results in the context of previous works and highlights the challenges of these groups and the highly morphological variability. The section is, again, a bit dense with long sentences that sometimes obscure the key messages. I am adding here some suggestions, but the text should be revised to make it more straightforward (e.g., avoid repetitions, short sentences, avoid repeating results).

Line 1274: I suggest reducing the sentence "...is polyphyletic, corroborating the revision by [22]." (more concise).

Line 1276- 1279: I suggest simplifying the sentence: "Representatives of the families Apataidae, Samlidae and Piseiontecidae form three highly supported and derived clades, not related to the 'core' group of Flabellinidae nor to each other (Fig. 1)."

Can you compare this with previous studies? If so, add reference.

Line 1279–1284: Rephrase to avoid repetitive language and simplify (this is already explained above) "We tentatively suggest synonymizing Unidentiidae and Piseinotecidae, but this should be reconsidered once molecular data for the type species *P. divae* become available".

Can you compare this with previous studies? If so, add reference.

Line 1286–1292: The sentence listing synapomorphies is long. Break into two sentences or eliminate redundant information as "of Samlidae lines 1289-1290).

Add references (I understand that have been already discussed refers to previous works).

I suggest rephrasing and simplifying "In the case of Samlidae we believe that" (Line 1291) to: "we consider that these synapomorphies have diagnostic value".

I consider that "and should facilitate the correct placement of newly described taxa even if molecular data" is redundant, that is the meaning of diagnostic value.

Line 1295–1297: I suggest emphasizing the unexpected nature of the relationship "...a possible sister relationship between Samlidae and Notaeolidiidae, an unexpected result given their markedly different morphologies."

Also change the order in which you are presenting the characters so you start with Samlidae.

No need to use connectors as "At the same time" to simplify sentences.

Line 1304–1307: Could be shorter as for example "...Contrary to previous studies, the expected sister relationship of Cumanotidae and Pseudovermidae was not recovered."

Line 1311 -1314: Rephrase to improve readability "The resulted relationships (Notaeolidiidae + Samlidae; Cumanotidae + Apataidae) should be considered as tentative, since our analysis lacks many aeolid taxa, including lineages of the large family Facelinidae s.l."

Line 1314: Eliminate "We also believe" from the sentence.

Line 1320-1323: This can be reduced by eliminating part of the sentence as you are already referring to the figure "~~At the same time~~, Moreover in all our phylogenetic analyses *Bajaeolis* is recovered in a clade including representatives of the family Flabellinopsidae~~: *Flabellinopsis iodinea* and *Baenopsis baetica* (the monotypic genera *Flabellinopsis* and *Baenopsis*, respectively)~~ (Fig. 1)"

Line 1329-1333: "At the same time" is used twice to connect sentences. Please revise the full text to avoid repetitions. (use e.g., Similarly)

Line 1336–1337: "In general" can be eliminated

Line 1366-1369 : "At the same time" is used twice to connect sentences. Please revise the full text to avoid repetitions. (use e.g., Similarly)

Line 1374: Change to "It should also be noted that" or even to be more concise as "Notably"

Line 1404–1412: Gramma is not correct, moreover I think this is important and could be clearer, e.g.: “Our analyses demonstrate that adding new taxa to the phylogenetic dataset required reassessing genus-level diagnostic characters in Coryphellidae and Flabellinidae sensu [22]”

Line 1415: No need to define synapomorphies

Line 1440-1441: I do not understand what you mean with “incorrect interpretation of characters”. In past studies? Please clarify.

Line 1441-1442: I suggest rephrasing as for example “Our results highlight the limitations of adopting a splitting approach in this context”

Line 1456: “Our study one more time demonstrates”, one more time? Are you referring to previous studies (add references if so)

Line 1458: Our study is repeated, please rephrase.

Line 1464: What do you mean with predictable?
